# Exploring the experiences of women living with metastatic breast cancer [MBC]: A systematic review of qualitative evidence

**DOI:** 10.1371/journal.pone.0296384

**Published:** 2024-01-05

**Authors:** Trína Lyons-Rahilly, Pauline Meskell, Eileen Carey, Elizabeth Meade, Donal O’ Sullivan, Alice Coffey

**Affiliations:** 1 Department of Nursing & Health Care Sciences, Munster Technological University, Tralee, Kerry, Ireland; 2 Department of Nursing & Midwifery, University of Limerick, Limerick, Ireland; 3 Oncology Department, HSE Dublin Mid Leinster, Midlands Regional Hospital, Tullamore, Co. Offaly, Ireland; 4 MTU Kerry Library, Munster Technological University, Tralee, Co Kerry, Ireland; The Chinese University of Hong Kong, HONG KONG

## Abstract

**Purpose:**

Metastatic breast cancer [MBC] is the leading cause of cancer death in women globally with no cure. Women diagnosed with MBC endure a catastrophic upheaval to multiple aspects of their life and a radically transformed future landscape. Evidence suggests that the provision of care for women living with metastatic breast cancer is inadequate, socially isolating and stigmatising. To date, this topic has received little research attention. To increase understanding of the experiences of women living with MBC, a synthesis of current evidence is required. This paper presents a review of qualitative evidence on women’s experiences of MBC.

**Methods:**

A qualitative evidence synthesis [QES] was conducted to synthesise primary qualitative research on the experiences of women living with MBC. Searches were performed of electronic databases Medline, Medline Ovid, PsycINFO, Psych articles, PubMED, CINAHL Complete, Scopus and grey literature databases. The methodological quality of the included studies was appraised using a modified version of the Critical Appraisal Skills Programme [CASP]. Title, abstract, and full-text screening were undertaken. A ‘best fit’ framework approach using the ARC [Adversity, Restoration, Compatibility] framework was used to guide data extraction and synthesis. Confidence in the findings was assessed using the Grading of Recommendations Assessment, Development and Evaluation, Confidence in the Evidence from Reviews of Qualitative research [GRADE-CERQual].

**Results:**

28 papers from 21 research studies containing 478 women’s experiences of living with MBC were deemed suitable for inclusion in this qualitative evidence synthesis. Findings are presented in a new conceptual framework RAAW [adapted from ARC] for women living with MBC under themes: **R**eality, **A**dversity, **A**djustment and **W**ellbeing. Findings revealed that a diagnosis of MBC impacted every aspect of women’s lives; this is different to a diagnosis of early breast cancer. An overarching theme of lack of support extended across various facets of their lives. A lack of psychological, emotional, and psychosocial support was evident, with a critical finding that models of care were not fit for purpose. Deficits included a lack of information, knowledge, inclusion in shared decision-making and MDT support, specifically the need for palliative care/oncology support access. Some women living with MBC wanted to be identified as having a chronic illness not a life-limiting illness. Culture and socioeconomic standing influenced the availability of various types of support. The impact of treatment and symptoms had an adverse effect on women’s quality of life and affected their ability to adjust.

**Conclusion:**

This review synthesised the qualitative literature on the experiences of women living with MBC. The ARC framework used in the synthesis was adapted to develop a revised conceptual framework titled **RAAW** to represent the evidence from this review on experiences for women living with MBC; **R**eality & **A**dversity: A diagnosis of MBC; **A**djustment: Living with MBC; **W**ellbeing: Awareness, meaning, engagement [RAAW; MBC].

## 1. Introduction

Breast cancer significantly impacts women’s lives physically, psychologically, and socially; in 2020, there were 685,000 deaths worldwide from breast cancer [1, 2]. Breast cancer is referred to as ‘metastatic’ or ‘advanced’ if it cannot be removed with surgery or has travelled to other sites within the body [3]. Metastatic breast cancer [MBC], including metastases found at diagnosis and recurrence post an early diagnosis of stage I-III, represents the most serious type of breast cancer [4]. With developments in treatment, women with metastatic breast cancer are living longer; consequently, it is essential to understand the experiences and needs of women with MBC to inform future models of care [5–7].

A qualitative evidence synthesis [QES] is the systematic gathering of primary qualitative data on similar topics of interest [8–10]. This maximises an understanding of health conditions, gleaning a deeper awareness of individuals’ experience of a disease, perceptions of their health condition, and decisions concerning their use of health service delivery [11]. QES can increase our understanding of cultural communities and has applicability within nursing as it offers a significant opportunity to develop knowledge. The QES process facilitates an uncovering of the deeper meaning of phenomena: it is not just a description of how people feel about a health condition, topic, or treatment but an understanding of ‘why’ they feel and behave the way they do [12].

### 1.1. Rationale for the study

Currently, there is a dearth of research into the experiences of women living with metastatic breast cancer [13]; Major gaps exist in the treatment and management of metastatic breast cancer patients, whilst the death rates due to breast cancer, are expected to almost double over the next 15 years, highlighting the crucial need to address these gaps [14]. The limited available literature on women living with MBC reveals high symptom burden, stigma, isolation, physical, psychological, psychosocial, and spiritual concerns with added financial pressure along with the associated complexity of decision-making [15–17]. Therefore, more challenging decisions must be made concerning women’s health and future care needs. It is recognised that these women require organised support that is different from women with an early breast cancer diagnosis [17–19]. MBC remains a virtually incurable disease, with a median survival time of two years, with some women living to five years or more [1, 17]. Moreover, MBC is associated with a humanistic and financial burden to individuals, families, carers, society and health care systems [1]. Further research is required on the experiences of different subgroups of women with MBC, particularly those who have lived with MBC long term, from different cultural communities, women living well with MBC, and those receiving different kinds of active or palliative treatment [13]. With developments in treatment, women with metastatic breast cancer are living longer; therefore, it is essential to understand the experiences and needs of this cohort of women [5–7]. Significant adjustment is required to current models of care, as these are no longer fit for purpose given the increasingly chronic nature of the disease. Women living with MBC engage with palliative care services only at the end-of-life juncture, this is a relatively short period compared to the length of the chronic MBC disease trajectory. It can be increasingly difficult to recognise when decline is occurring. An amalgamated palliative care and oncology approach is needed to support women and oncologists in making difficult decisions, such as when to stop treatment and to plan for end of life [20]. To date published research is fragmented, it is now timely to undertake a comprehensive synthesis of current evidence, to increase understanding of the experiences of women living with MBC, and to inform future models of care. To the reviewer’s knowledge no qualitative evidence synthesis current or underway has been completed to date.

This study presents a systematic review and synthesis of qualitative evidence of the lived experience of metastatic breast cancer. It was conducted to evaluate and synthesise qualitative evidence, exploring women’s attitudes, perceptions and experiences regarding living with MBC. The method encompasses a comprehensive search for retrieval of qualitative research publications, a critical appraisal of primary qualitative studies, classification of results and synthesis of key findings. This qualitative evidence synthesis facilitates the identification of the issues women encounter living with MBC. This information can be used to inform the development of care trajectories to optimise care. In addition, this qualitative evidence synthesis will contribute significantly to understanding the needs of these women and facilitate the development of policy and practice to support their needs.

## 2. Aim

This QES aimed to synthesise evidence from principal qualitative studies, exploring women’s attitudes perceptions, and experiences of MBC, and establish an aggregated holistic view of the overarching themes related to this experience [21].

### 2.1. The research question is

What are the attitudes, perspectives, and experiences of women living with metastatic breast cancer?

### 2.2. The objective

The objective of this Qualitative Evidence Synthesis is to synthesise primary qualitative research exploring women’s attitudes, perceptions, and experiences regarding living with metastatic breast cancer.

### 2.3. The topic of interest

The phenomena of interest in this review are attitudes, perspectives, and experiences of women living with metastatic breast cancer.

## 3. Method

**Fig 1:**
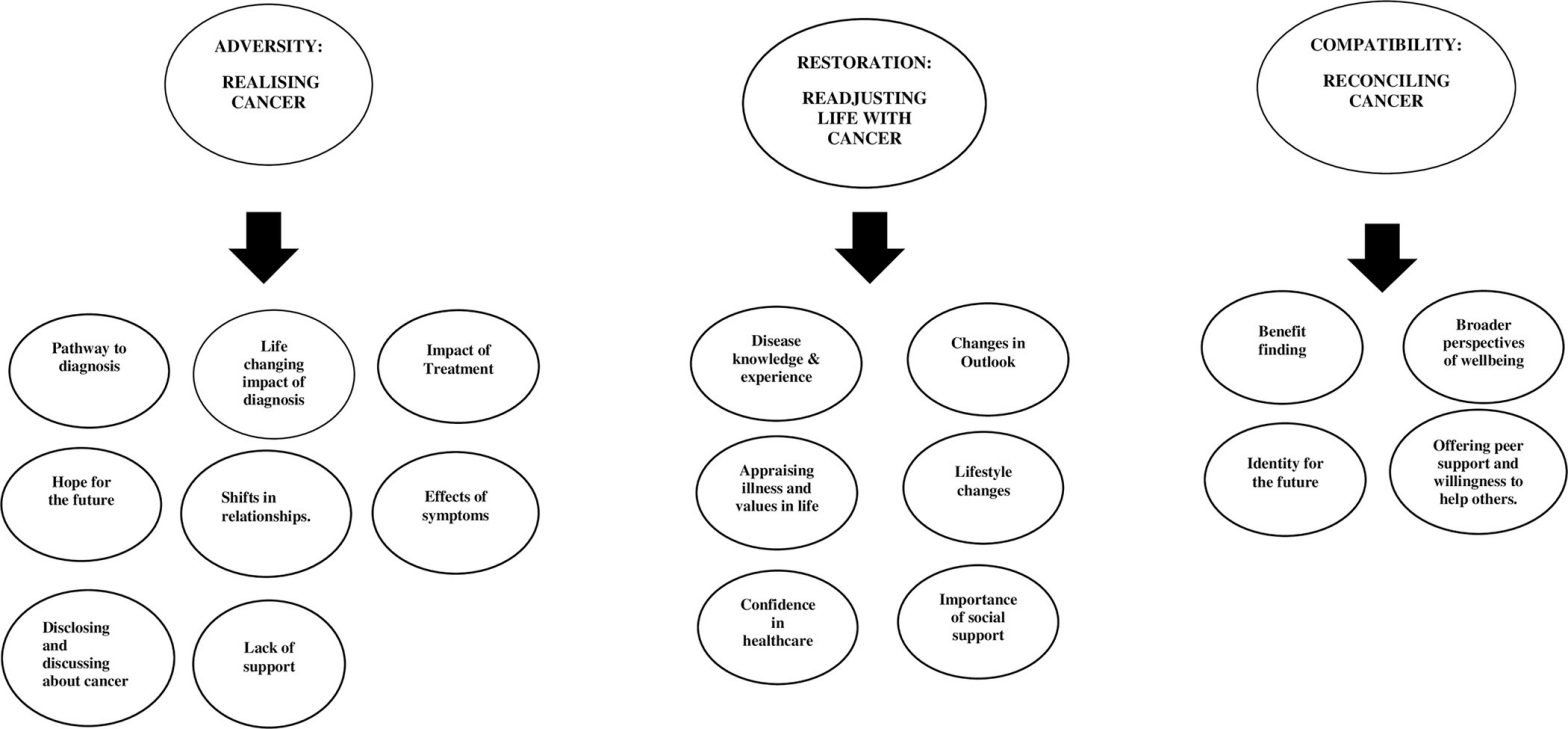
The ARC [Adversity, Restoration, Compatibility] conceptual framework for individuals living with and beyond cancer [24].

A protocol on this QES was developed by applying Cochrane Effective Practice Organisation of Care Group [EPOC] guidelines template. The protocol was registered with Open Science Framework [22]. The ‘best-fit’ framework approach to synthesis was chosen as the most suitable methodology to undertake this synthesis. S1 **Table** outlines the seven steps of the ‘best fit’ framework technique undertaken [23]. Framework synthesis is better used when an a priori framework can be applied to the review therefore, the concepts that drive the framework are secure beforehand [10]. The ARC [Adversity, Restoration, Compatibility] conceptual framework for individuals living with and beyond cancer [24] was adopted in this QES [**Fig 1**]. The ‘best fit’ framework synthesis procedure allows testing, and reinforcement building on an established model for a similar population [10, 25].

### 3.1. Systematic identification of primary qualitative studies

The Spice Framework **S2 Table** was used to identify the main concepts of the review focus, classify key search terms for the search string, and conduct a search for theoretical frameworks to be used as the ‘best fit’ framework. The inclusion and exclusion criteria identified keywords and search strings were agreed. Criteria for inclusion and exclusion for this search can be reviewed in S3 **Table**. A summary of the electronic search strings is presented in S4 **Table**.

### 3.2. Search strategy

With the assistance of a Librarian [D O’S], a systematic search for primary qualitative research studies was undertaken on Medline, Medline Ovid, PsycINFO, Psych articles, PubMed, CINAHL Complete, Scopus and grey literature databases. This search was undertaken from January 2010 to July 2020 and a further updated search using the same search strategy was undertaken from July 2020 to December 2022. The reference lists of all the included studies and key references [i.e. relevant systematic reviews] were checked for any salient studies not identified in the original search. Using the same keywords, a separate grey literature search was undertaken to identify primary qualitative literature not indexed in the databases listed, this was conducted on the following sources:

- Open Grey [www.opengrey.eu]
- Grey Literature Report [New York Academy of Medicine; www.greylit.org]
- Agency for Healthcare Research and Quality AHRQ [www.ahrq.gov]
- National Institute for Health and Clinical Excellence [NICE; www.nice.org.uk]: http://www.eldis.org.
- Bielefeld Academic Search Engine [BASE]

**Fig 2:**
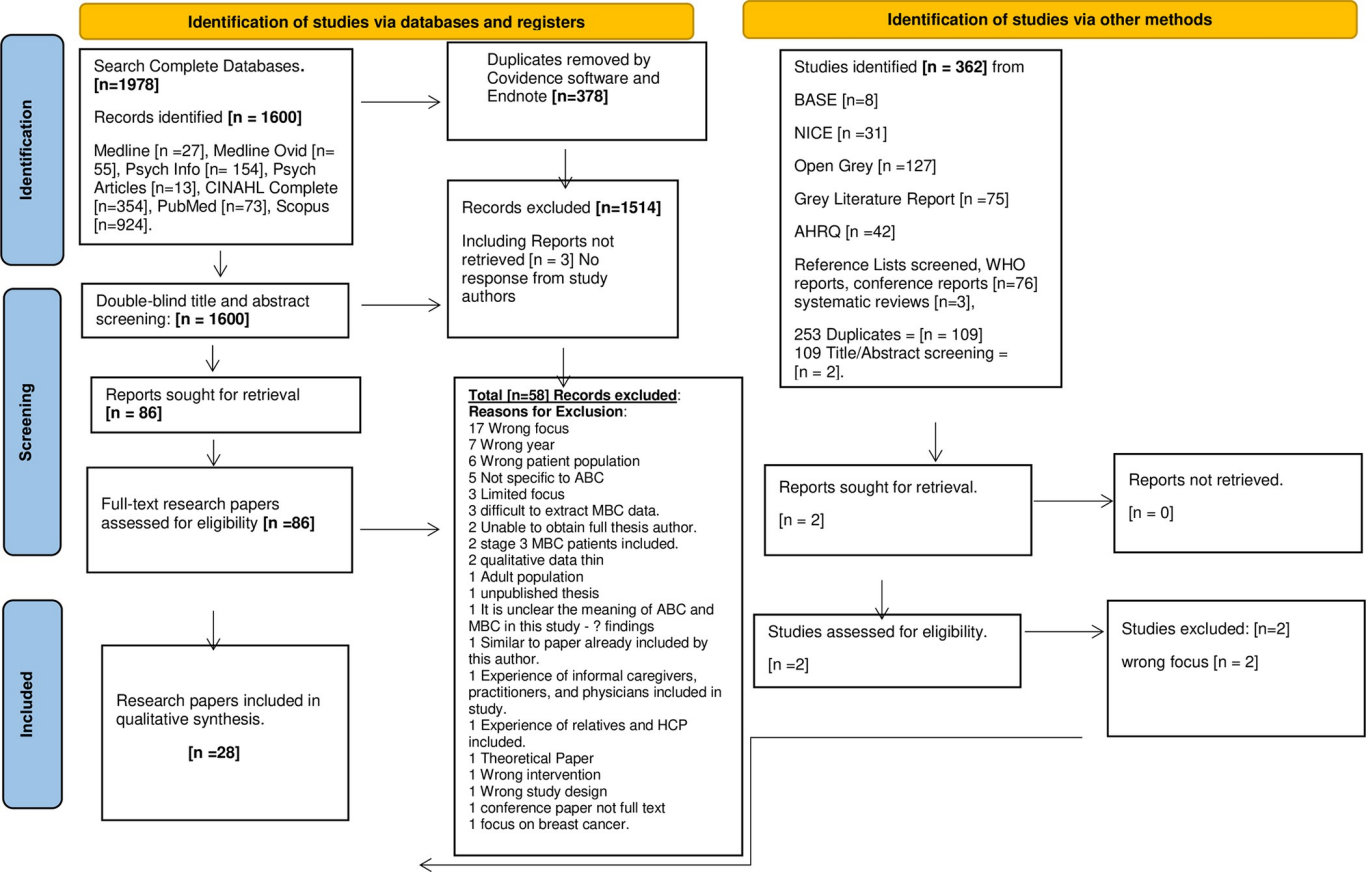
PRISMA flow diagram.

Both comprehensive literature searches have been combined from January 2010 to December 2022 and reported in the PRISMA flow diagram [**Fig 2],** identified studies were collated and uploaded to EndNote X9 [26]. The search outputs from of 1978 research papers were imported into Covidence, [27] a systematic review software package for the management of reviews; 378 duplicates were removed. Utilising this software accommodates filing the imported references accordingly [28]. The lead reviewer [TLR] the supervisory team [AC, PM, EC], and an Advanced Nurse Practitioner in Oncology [LM] undertook double-blind title abstract screening of all 1600 research papers. 1514 studies were deemed irrelevant. Three study authors were contacted to gain permission to access their research papers, no response was received, so these studies were excluded. Disagreements were resolved between team members. This resulted in a full-text review of 86 research papers. Following the full-text screen review, 28 papers were selected for inclusion. Justification for excluding fifty-eight full-text papers that did not meet the inclusion criteria are presented in [**Fig 2]** PRISMA. There are 28 papers from 21 research studies included in this QES, details of which are summarised in S5 **Table** **characteristics of included studies.** The literature search results are reported using the PRISMA flow diagram [29, 30] **[Fig 2].**

### 3.3. Quality appraisal of included studies

A quality appraisal was performed employing an adapted qualitative Critical Appraisal Skills Programme [CASP] checklist/tool [31], S6 **Table**. TLR undertook the assessment, assessing methodological limitations using the CASP tool. This facilitated identifying and categorising quality aspects of research conduct and reporting of included studies. This resulted in richer quality papers contributing more to the synthesis as opposed to those of lower quality [32]. The justification for using this appraisal was not to eliminate poor-quality papers but to decipher the quality of studies relative to their potential influence on findings [33]. TLR and PM conducted a pilot of the CASP on three studies to test the tool itself and its application. Once pilot evaluations were completed, TLR, PM, AC and EC discussed, and compared findings and a consensus was reached regarding the tool’s application and interpretation [34].

### 3.4. Selection of a framework for ‘best fit’ analysis

Selecting a theory/framework for ‘best-fit’ synthesis was conducted using the BeHEMoTh [Behaviour of Interest, Health context, Exclusions & Models or Theories] technique [23]. This technique presents a viable effective method for identifying a theory for a systematic review [23]. It is important to note it is not a requirement for the theoretical framework to be an ideal fit for the question [35].

## 4. ‘Best Fit’ framework synthesis

The ‘best fit’ framework synthesis method employing the ARC framework was utilised in developing the synthesis for this review [36]. This process is outlined in seven steps in S1 **Table** [23]. This approach can be tailored towards patient experiences of a particular disease trajectory [25], such as women’s experience of living with MBC. It offers an opportunity to underpin and shape existing published models [36]. It is better adapted to research with a specific question, where the prime concern is to define and understand what is occurring within a particular setting or patient’s experience of a specific disease trajectory [25]. This QES comprises of 28 papers from 21 research studies. These papers were examined to identify theoretical conceptual models employed. A search of databases was also conducted using the BeHEMoTh template to review suitable theories/frameworks and to engender a priori framework, to code the evidence against. The Adversity, Restoration, Compatibility [ARC] conceptual framework, for living with and beyond cancer, developed by Le Boutillier et al. [24], was identified as the most suitable framework to use in this review. Thus, an augmentative deductive process was undertaken, building on this existing model [36].

### 4.1. The ARC conceptual framework

The resulting overarching ARC framework [**Fig 1]** represents Adversity [realising cancer], Restoration [readjusting to life with cancer] and Compatibility [reconciling cancer]. The themes are interlinked because the experience of living with and beyond cancer is not one dimensional. The ARC framework was established from the lived individual experience of the person affected by cancer. It is also a more suitable model than chronic illness models, as it is patient-centred, focusing on living with and beyond cancer [24]. ARC themes support biographical disruption, coping strategies in managing disease, attitudes to loss of self and go beyond the physical suffering of illness. Furthermore, Le Boutillier et al. stipulate that the framework is patient-centred and valuable in shaping supportive clinical oncology care [24].

### 4.2. Data extraction

The ARC framework was populated within a specifically adapted google form, this was devised by one team member PM. TLR became familiar with the 28 papers. PM, AC, EC undertook indexing of four studies to ensure transparency. During the process of indexing TLR read the papers line by line and populated the google form with the data from the 28 primary papers. This was undertaken in a deductive manner, while being mindful of not forcing data that did not ‘fit’ into unsuited categories [10]. Following this, outstanding data was synthesized inductively using the approach of thematic synthesis [33] to develop themes until all the data were accounted for [37]. Therefore, a specific two-phase subsequent method to ‘best fit’ framework synthesis facilitates an audit trail of themes evolving from the framework synthesis and those from the subsequent thematic synthesis [37]. The ‘best fit’ framework synthesis technique follows seven steps [23] [S1 **Table**]. The 28 papers included were mapped to the adjacent themes resonating with each framework category. As synthesis progressed, new themes emerged. The framework was adapted to better reflect a conceptual framework specific to women living with MBC. This process allowed for the identification of a gap in knowledge pertaining to the experiences of this cohort of women.

### 4.3. Data synthesis

The data synthesis of the qualitative studies and evolving findings were continually discussed in supervisory team meetings to interrogate the data and enrich the synthesis. The a priori categories in the identified model were used to guide the synthesis in a series of five stages:

**Identifying a thematic framework:** An a priori framework, the ARC framework [24], was utilised for qualitative data extraction to guide the synthesis, the framework was adapted and constructed on the emergent themes from the analysis.

**Familiarisation:** TLR became familiar with the data by reading the included primary research papers, fluidly moving over and back reviewing the data and the themes identified in the studies. The conceptual themes of the ARC framework were assimilated within an adapted google form.

**Indexing:** Indexing involved TLR applying the ARC Framework to code each of the papers. This was achieved by TLR extracting data from the research papers and mapping it against the ARC framework. Data coding was undertaken based on the themes identified in the data. Each paper was indexed using the codes associated to the themes of the framework. During this process TLR became immersed within the 28 studies and continuously moved between the data, developing themes and discussing emerging data/themes with AC, PM, EC and LM. All papers were read and reviewed until there was a consensus there were no new emerging themes. TLR presented findings of the data extracted via the ARC framework on google forms on a MIRO diagram to the team [AC, PM, EC, & LM]. In-depth discussion and debate with the team took place and revisions were undertaken.

**Charting/Mapping:** Charting/mapping involved rearranging data according to the relationships between themes, mapping and interpreting data where the range and nature of reviewed concepts were mapped and associations between themes identified. The review question and aim was maintained in focus throughout this process. The data was sorted by theme and presented in the form of an analysis. The columns rows of the table reflect the studies related to themes and allowed comparison on findings of the research papers across different themes, subthemes and subsequently new themes developed from the evidence in identified studies.

**Fig 3:**
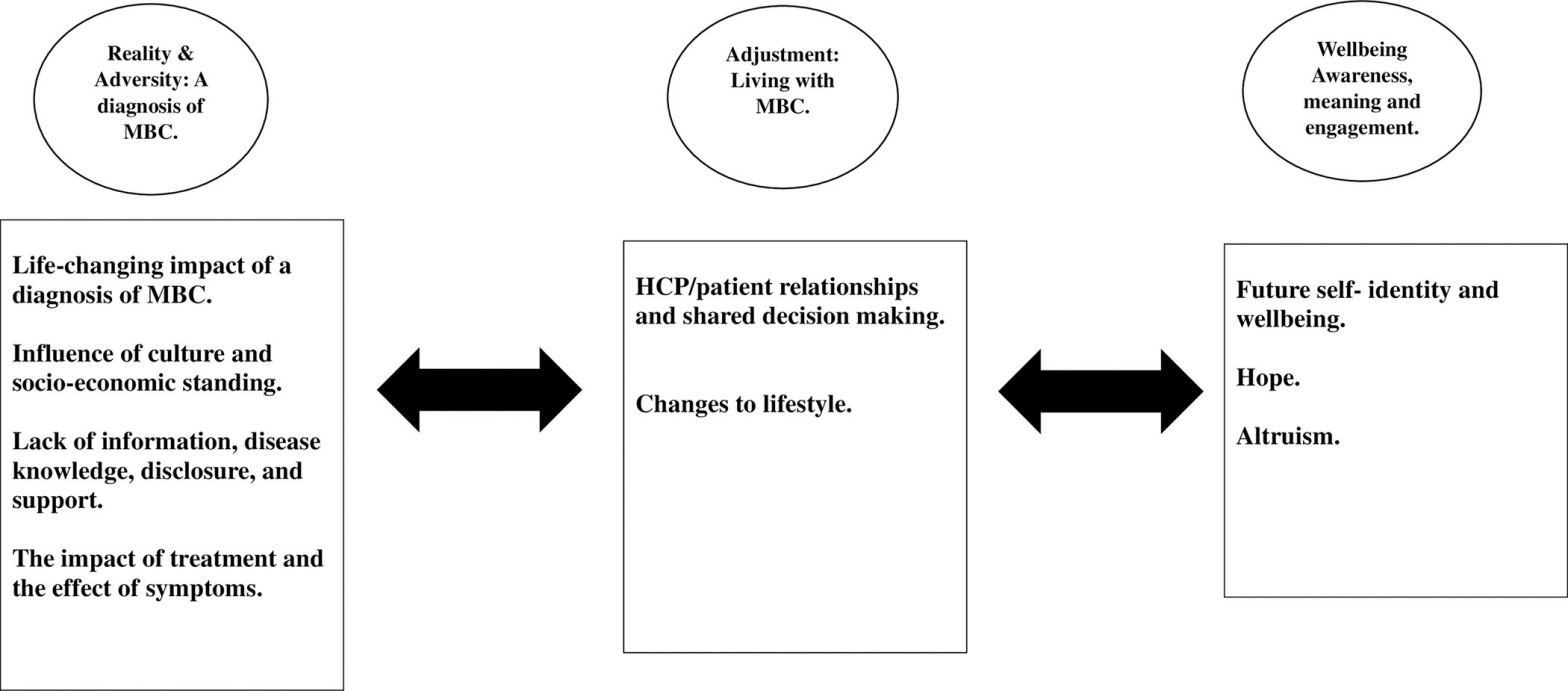
RAAW: The conceptual framework for women living with MBC [adapted from ARC]. **R**eality & **A**dversity: A diagnosis of MBC; **A**djustment: Living with MBC; **W**ellbeing: Awareness, meaning engagement [RAAW; MBC].

As outlined an a priori framework, the ARC framework [24] was utilised for qualitative data extraction to guide the synthesis. The framework was adapted based on the emerging themes from the analysis. As further concepts developed during synthesis that did not translate to the prevailing themes, thematic synthesis was undertaken to create new themes that were built/added onto the existing framework. This took time, TLR continuously reviewed emergent themes and concepts in partnership with AC, PM, EC and LM. The framework was adapted accordingly, established from the existing conceptual ARC framework encompassing the new concepts and theories [36]. This is a non-linear framework, the arrows represent the oscillations within the MBC disease trajectory. The experience of living with MBC does not remain static, there are fluctuations across the framework. This new non-linear framework **[Fig 3]** is named **RAAW**: The Conceptual Framework for women living with MBC [adapted from ARC]. **R**eality & **A**dversity: A diagnosis of MBC; **A**djustment: Living with MBC; **W**ellbeing: Awareness, meaning and engagement [RAAW; MBC]. It is the researcher’s responsibility to ensure that the context of the qualitative primary research data is not misconstrued during the extraction synthesis process; TLR was cognisant of this throughout the process [34]. Enhancing the transparency in reporting the synthesis of qualitative research [ENTREQ] for this review can be viewed in **S8 Table**.

## 5. Results

### 5.1. Synthesis output

The findings of the synthesis are presented under the following themes.

**R**eality and **A**dversity: A diagnosis of MBC,

**A**djustment: Living with MBC,

**W**ellbeing: Awareness, meaning and engagement [**RAAW**; MBC].

These represent **RAAW:** The Conceptual Framework for women living with MBC [adapted from ARC]. **R**eality & **A**dversity: A diagnosis of MBC; **A**djustment: Living with MBC; **W**ellbeing: Awareness, meaning engagement [RAAW; MBC] [Fig 3].

### 5.2. Reality and adversity: A diagnosis of MBC

This is one of three main themes divided into four associated subthemes. Adversity for women living with MBC related to the initial shock and fear of cancer recurrence, the reality that they now live with a life-limiting condition.

#### 5.2.1 Life changing impact of a diagnosis of MBC

From the moment of their initial diagnosis of breast cancer, to the juncture of diagnosis of progressive MBC disease, many women lived for an extended time with the prevailing thoughts and fears that cancer may return; it was always in their consciousness [6, 38]. The news that breast cancer had progressed was profoundly shocking and terrifying. Women described being fearful for the future and reported a loss of hope, heightened uncertainty, and reduced control over their lives [39, 40]. This was noticeably different from the women’s reactions to previous initial diagnosis with primary breast cancer, as at that point there was a potential cure, whereas with MBC, the focus was not on cure but on extension of life [41–44]. The women revealed that progression of the disease, debilitating symptoms of treatment, the side-effects of previous surgery frequently led to reduced functional ability, psychological distress, physical discomfort and ultimately altered body image [41, 45–47].

The diagnosis had a damaging impact on their lives and precipitated a sense of loss of the women’s holistic existence, affecting their professional identity. For example, many women had to give up work [38]. Worries about personal appearance; relationships with partners; role as mothers; bonding with family and friends; loss of purpose, well-being, and interference with the ability to manage activities of daily living were reported as overwhelming [41–43, 46–50].

#### 5.2.2 Influence of culture and socio-economic standing

Drawing on data from studies conducted in Africa, Malaysia and two from the USA, a new subtheme emerged relating to cultural socio-economic influences on the reality of living with MBC [51–53]. These women had a significant financial burden and a perceived health provider bias related to their lower socio-status, race, and ethnicity. This resulted in women having little trust in the medics caring for them, significantly reducing their quality of life. This has highlighted that equitable comprehensive end-of-life care needs to be available to all, no matter the socio-economic status [53]. Breast cancer is increasing in developing countries, where women are presenting later with more advanced disease. There is a requirement for these women to be educated on the signs of early detection of breast cancer, this would allow women to be empowered to make decisions on their care [48].

#### 5.2.3 Lack of information, disease knowledge, disclosure, and support

When diagnosed with MBC women became active self-managers of their care searching for reliable, relevant information. However, gaining knowledge on their disease was difficult as women had limited access to literature and struggled to get information/supports from medical professionals [41, 54–56]. Women regularly turned to social media to explore support options and learn more about the disease treatment [54]. On initial diagnosis there was support for women from a physical point of view, but this did little to meet their psychological needs [20]. Moreover, the most unmet need centred on a lack of psychological counselling and information [40, 56]. Conversations and good communication with healthcare professionals were indicated as being integral to forming a supportive network, this was often lacking, as demonstrated in eight studies [40, 41, 43, 47, 50, 55, 57, 58]. Women had negative experiences of being talked about by Health Care Professionals (HCP) while still in the room. They were treated as if they did not understand the medical terminology used and felt unsupported in dialogue about living with this disease [55]. Additionally, there is a deficiency in accessibility to clinical trials for women with MBC and difficulty in obtaining information on their disease evolution. There is a need to have a specialised MBC nurse whose role could potentially support women with MBC throughout their cancer trajectory [38, 59]. Furthermore, there was a lack of conversation from HCPs on aspects of the disease that affected women’s sexuality because of treatments [47].

There is a clear differentiation between those diagnosed with early breast cancer compared to those living with metastatic breast cancer [17–19]. These two disease trajectories are often intertwined but are, however, very different. This led women to feel forgotten about and accentuated this sense of silence that had already been imposed upon them. Women stated that their experience deserved to be recognised [60].

In a longitudinal mixed methods study by Reed and Corner, few women appeared to have access to official support services, describing their primary support as family and friends [20]. For women living with MBC the primary source of meaning in life was family and valued social relationships; women acknowledged that social support played a pivotal role in living well [40, 49, 51, 53, 58]. Women with MBC drew mainly on family for support; however, few supportive formal resources are available to families, which often resulted in an additional burden on family members assisting women with MBC [40, 56]. Women with MBC were constantly striving for normality through communications with a select group of friends or inner circle; these individuals were confidants and an important social espousal for women with MBC [41, 42, 55]. This was a form of self-management on the women’s part, creating a new selected supportive network. As the disease progressed, the role of the select group of family and friends also had to evolve [61]. Children were often the ‘silent observers’ of the disease trajectory; roles were reversed with the children looking after mothers; women felt it easier not to talk about their diagnosis to keep an equilibrium in the household [41, 43]. They wanted specialised support for their children and partners [6, 47]. Being a mother placed women with MBC in the position where the needs of their family and children were often placed before their own [57].

Furthermore, talking about their diagnosis of MBC was challenging. Women were selective in their dialogue with others. There was a delicate balance between preserving privacy on the aspects of living with the disease and getting support [41]. Women tended to take control of their illnesses to safeguard themselves by being conservative in conversations about cancer and were cautious with whom they shared information [49, 54, 57]. Because women with MBC looked ‘normal’, there was a heightened lack of awareness and sometimes disbelief that women were living with MBC [54]. In a cross-sectional qualitative study [42] and an interpretative phenomenology analysis [IPA] study [60] women used tactics called ‘strategizing disclosure’. This involved discussing specific elements of their diagnosis and health with family and friends. This led to suffering in silence for women as they could not verbalise their feelings around the disease trajectory. Approaches to dialogue could be categorised into 1] selective on who to tell, 2] selective on what to tell, 3] avoiding negative discussions, 4] personal feelings of anger and resentment.

Women reported that they did not attend a support group because they felt most women in support groups they attended had an early-stage cancer diagnosis rather than MBC, they could not relate to this [20, 55, 56]. Women expressed a need for support groups with women of a similar age and stage of diagnosis [47, 58]. This subtheme relates to the paucity of information and discourse in the area of MBC. It includes a lack of medical support, shared decision-making, information/disease knowledge, poor psychological support, an absence of MBC specific support groups for women and highlights the importance of the support of family and friends. Furthermore, there is a need to have a specialised breast care nurse whose role could potentially support women with MBC throughout their cancer disease trajectory [38, 59].

#### 5.2.4 The impact of treatment and effect of symptoms

Treatment was defined as the ultimate suffering [4], with women with MBC unable to fully articulate the experience. Women had constant concerns about whether the treatment was working, if there were other treatment options and if the side effects of these treatments could potentially affect their activities of daily living [38, 40, 49, 57]. In three studies, women experiencing MBC choose to have the most aggressive form of treatment for the longevity of life—stopping the treatment was not an option [40, 50, 51]. Women were determined to cope with debilitating side-effects that impacted every system in the body, as it meant prolonging life; their days were planned around this treatment [50]. Additionally, living on borrowed time caused enormous psychological stress, which included being hyper-alert to symptoms of possible relapse. Women desired continuity of care within the multidisciplinary team; they wanted to meet the same HCP’s, which enabled good collaboration, communication and understanding of treatment planning. Women wanted to reduce the time spent in treatment and attending clinical appointments; significantly, the women in this study wanted to focus on living, not simply surviving [40].

Women with MBC had various debilitating symptoms from treatment that diminished their quality of life. These included neuropathy, pain, hair loss, sores, fatigue, breaking bones, nausea, chemo brain, constipation, diarrhoea, oral mucositis, anorexia, inability to mobilise, vaginal dryness, chemo-generated menopause and low libido [39, 42, 47, 48, 55]. Decreased functional status forced women to modify how they engaged with everyday activities, such as housework, self-care, family life, rest and food preparation [46]. The essence of coping was complying with the treatment offered [51]. Aspects of having to continue to work to maintain health insurance, despite feeling unwell, had far-reaching consequences [53]. Insufficient financial resources sometimes forced a delay in necessary treatment, thus raising the risk of inadequate symptom management and premature mortality [53]. Psychological loss, grief and anxiety characterised the emotional landscape. Women found the emotional challenges combined with a reduced capacity to make decisions and the realisation that they would be on treatment for the remainder of their lives overwhelming [47, 54].

## 6. ‘Adjustment’—Living with metastatic breast cancer

The second central theme, ‘Adjustment’ to living with MBC, refers to women with MBC adapting to life in the context of living and managing MBC disease. During this phase of the disease trajectory, women adjust to living with a new normal and navigating the intricacies of life that come with a metastatic breast cancer diagnosis.

### 6.1. Health care professionals [HCP]/patient relationships and shared decision making

The supportive scaffold for women adapting to the new normal of living with MBC was their relationship with the HCP and the wider multidisciplinary team [MDT] [62]. The importance of clarity in autonomously making shared decisions was paramount, and the need for recognition of others’ expertise within the multidisciplinary team [47]. There is a discrepancy between various treatment programmes, with the ultimate focus on longevity rather than the quality of life [49, 55]. Evidence in two studies depicted the overarching concept of the complex relationship women had with their oncologists and how this relationship impacted women’s healthcare outcomes and decision-making [20, 50].

The relationship women had with their Doctors/HCP’s was linked to enabling women to make informed choices, become involved in shared decision-making, and have greater satisfaction in the care they received, thus improving their quality of life [39]. Fourteen studies referred to this subtheme and focused on the importance of knowledgeable shared decision making for women with MBC; using easily understood language; it underlined the need for an open, honest relationship with doctors/HCPs and the wider MDT in charge of women’s care. These studies further emphasised the importance of educating women on the transitions within the metastatic breast cancer disease trajectory and the necessity of including the palliative care experts and the broader multidisciplinary team to allow these women to make informed autonomous decisions about their care trajectory [20, 40, 41, 44, 47, 50, 51, 53, 55, 59–63].

### 6.2. Changes to lifestyle

Women made various lifestyle changes due to altered functional status, debilitating symptoms, and side-effects of treatment. Various creative lifestyle changes were adopted, such as painting, mindfulness, and an interest in nutrition. One of the main reasons for a lifestyle change for women with MBC was the exacerbation of treatment and symptom burden, which restricted women’s lifestyle, functional status, and disrupted activities of daily living [48, 49]. Furthermore, concentration difficulties negatively affected relationships with others, and changes to their body adversely impacted their self-image [39–41, 43].

The capacity for women with MBC to utilise strategies to live well was explored in three studies [49, 53, 61]. Findings indicated that women differed in the methods they employed to live well. The most common strategy to live well was to restore a level of normality within their lives; all these women had children [49]. The second strategy was to reevaluate their lives; they used exercise to improve quality of life, reduced stress, having a positive attitude, and social support were vital in attaining this. However, due to fluctuating health status from treatment symptoms, restrictions to lifestyle led to burden, poor quality of life, and a loss of a sense of purpose to participate fully in social roles.

## 7. Wellbeing: Awareness, meaning and engagement

The theme wellbeing: awareness, meaning, and engagement refer to women finding a way to face the future with a renewed outlook on their lives, a new sense of personal worth and a sense of empowerment to live well. This theme is constructed on the subthemes of future self-identity and wellbeing, hope and altruism.

### 7.1. Future self-identity and wellbeing

A realisation that women with MBC were living on reduced time appeared to be the incentive for a significant shift and change in how they lived their everyday lives, particularly time spent with families. Initially, women felt a multitude of emotions at diagnosis of MBC, such as fear, dread and anxiety; However, as time passed, a sense of acceptance allowed the women to gain an appreciation for life; in facing their mortality, time left was perceived as a gift [53]. This allowed them to gain control of their life and achieve a positive mindset [49]. Women perceived their prognosis as uncontrollable; they wanted to control what they could, and having a positive mindset played a significant role in achieving a good quality of life [45, 61]. This involved adjusting perspectives to a new norm, concentrating on what was most important in life [46]. Key sources of meaning were children, family, selective close social relationships, spirituality, a new value of life, maintaining normality, and accepting the diagnosis [46, 50, 54]. Women began to live a more existential authentic life where beliefs and ethical values came to the fore and death became more meaningful [60].

Looking to an uncertain future, women living with MBC wanted to be identified as individuals living with a chronic disease instead of incurable cancer; they rejected the identity of being sick [38, 40, 49]. Planning a future for women with MBC is difficult as the complexity of the holistic experience of this illness leads to a ‘crisis of the presence’ this relates to losing oneself and ones place in the world [38]. Women with MBC reported losing their sense of femininity and were challenged to live with an altered body image [45, 48]. Working formed a significant aspect of their self-identity and allowed the women some normality to their lives [54]. The establishment of professional roles was a priority for some women and was an integral part of their identity. Women who were unable to work reported a sense of ‘valuelessness’; this, coupled with financial challenges, was difficult [42, 44, 47]. The women lived with an ‘altered mode of being’ they felt a disconnectedness from the world they lived in [60].

Living a life of engagement, purpose and meaning were integral to the wellbeing of these women [49]. Aspects of sexual health, mental wellbeing, psychological support were fundamental to maintaining wellness [45, 49, 63]. However, six studies allude to the enormous psychological stress that women with MBC experience, the palpable lack of support is coupled with a lack of research on interventions to help women cope [40–42, 47, 50, 56]. Furthermore, HCPs were not always qualified to support these women from this standpoint [59]. Yoga, painting, meditation, mindfulness, and the role of complementary therapies [42] were all considered helpful; however, these were instigated by the women themselves in these studies, not by HCP.

### 7.2. Hope

Hope in the midst of living with MBC was evident amongst women in the following studies [6, 20, 38, 40–42, 48–55, 61, 62]. Hope was defined differently for many women in eight studies [38, 42, 48–50, 52, 61, 62]. In these studies, women sought hope in the treatment they were receiving, in spirituality, a new outlook, living in the present moment, having a focus of short quantifiable goals, support from the multidisciplinary team, complementary therapies, and viewing their disease as a chronic illness, all of these brought hope and assisted the women to cope. Women searched for meaning in their suffering, [45] and found hope in adversity [48].

### 7.3. Altruism

In response to their personal experiences, numerous women felt encouraged to support other women with cancer and improve their understanding [53]. Altruism included a willingness for women to share their stories and to assist others in benefiting from the knowledge they had gained [47, 51, 54, 57]. These women promoted breast cancer awareness and helped others in a similar situation [51].

The women viewed life as living rather than just surviving and engaged in self-management methods that positively impacted their health. Therefore, they felt enabled to educate/inform women going through similar experiences [60].

## 8. Confidence in the evidence from reviews of qualitative research [GRADE-CERQual]

GRADE- CERQual allows for clarity and aids in evaluating confidence in the findings from a QES review [64, 65]. This approach evaluated the 10 review findings of this QES. These findings were derived from undertaking data synthesis applying the ‘best fit’ framework approach of the 28 papers. GRADE-CERqual is a systematic, robust framework allowing assessment of the confidence level in the findings of a qualitative evidence synthesis [65]. It is based on four components methodological limitations, coherence, adequacy of data and relevance. Two team members [PM, TLR] assessed the overall confidence in each of the review findings. The goal when assessing methodological limitations is to stipulate concerns when methodological limitations have been detected as significant to reduce the confidence in the finding [66]. Concerns regarding any component was discussed with the other two team members [AC, EC] and a consensus made. Confidence was judged as high, moderate, or low. The final assessment was based on consensus among the team [TLR, AC, PM, EC]. All findings started as high confidence and were then graded down if there were concerns. After assessing each component in relation to the total confidence in the evidence associated with each finding within this review, the supervisory team and the lead researcher made a judgment. A judgement is made in relation to concerns [66]. There are ten findings from this review; five were rated as high confidence, four were considered to have moderate confidence, one finding had low confidence. For more in-depth detail the results of the GRADE-CERqual are in S7 **Table** summary of qualitative findings table.

## 9. Discussion

The aim of this QES is to review the literature on the experiences of women living with metastatic breast cancer. The comprehensive data search yielded 28 papers from twenty-one research studies; it is apparent that there are clear correlations between the literature reviewed and the ARC framework. However, the evidence across studies indicates various unmet physical, social, and psychological needs and health care disparities for this cohort of women that were not represented within the framework. There is a consensus that the majority of women with MBC are living with a chronic illness; therefore, they will experience various oscillations within the disease journey [61]. The synthesised evidence from this QES highlights a considerable gap in the knowledge on how women with MBC live their lives daily and what approaches they use to manage their illness journey [49, 55, 57].

In particular a common theme throughout the literature refers to the psychological issues, emotional distress, and a lack of psychosocial support that these women are confronted with on a regular basis, usually cascading from the physical impact of treatment, symptom burden and underlying reality of transience of life [40–42, 45, 50, 55, 60]. The most challenging of these symptoms was pain, which is interrelated with fatigue, being anxious and depressed, this led to death anxiety, especially when feeling poorly [40, 50]. Coping with MBC necessitates relentless adaptation and adjustment [40]; women identified these transitions as adjustments in emotional, social, and physical wellbeing [61]. Unfortunately, fluctuations between illness, treatments and recovery undermined their ability to adjust. What is not evident within the literature is how these women overcame and moved through these various transitions throughout the disease trajectory.

These emotional transitions are periods of enormous psychological turbulence [40]. However, only two studies referred briefly to women’s mental health [42, 56] highlighting a gap in research. There is minimal uptake of mental health services amongst women with MBC, and more discussions are required with HCP’s around the mental health of these women [42, 56]. Women lacked a collaborative multidisciplinary approach to their care [37]. They were medically well treated however, the most significant unmet need was psychological support interventions [40]. Women used creative activities such as painting, art, and yoga as a form of distraction from emotional distress [58]. Although suggestions were made for women to utilise acceptance commitment therapy, cognitive behaviour therapy [CBT], and cognitive processing theory to enhance psychological wellbeing, there was no elaboration on how these could be undertaken [41, 45].

The literature also underscores a lack of supportive care services for these women [41]. There is an obligation to have a more synchronised delivery of care; this would be in the approach of a person-centred care plan and care services [41]. Women with MBC were often bewildered as to which multidisciplinary team member managed specific aspects of their care. A limitation in an integrated approach to care management emphasises the need for supportive nursing care services to facilitate the behavioural cognitive transitions experienced by women with MBC [46, 59]. This reflects the iterative aspect of the ARC framework where the women’s experience of MBC is not linear; they tend to transition from one phase to the next and back again depending on overarching factors such as treatment, symptoms, diagnosis, psychological wellbeing, time in the disease journey, relationships with HCPs and social relationships. Women with MBC require a person-centred approach to care that reflects their individual needs and challenges; HCPs need to be educated to assess these women individually and understand their perceptions of their symptoms and overarching concerns, all of which are personal to each woman [45, 59].

Models of care for women with MBC are no longer fit for purpose [20, 55] results from this QES support this. MBC is currently considered a chronic illness rather than a life-limiting disease, therefore arguably there is a requirement for utilising chronic illness models of care that favour self-management of care, for women living with an MBC diagnosis [20]. Additionally, HCPs need to recognise, identify, and facilitate when a woman is about to go through a significant transition and review/revisit their preferences for managing their care at these junctures. Breast care nurse specialists felt they lacked sufficient expertise, competencies, and time to deliver the degree of care necessary to women with MBC [55, 59, 67]. Despite the passage of time the situation has not improved, and breast care nurse specialists still need further training and education in MBC disease transitions and more clinical time to engage with this cohort of women. Specialist services appear not to be meeting women’s needs. Women with MBC reported unrecognised and unmet supportive care needs, supportive care from specialist nurses was ad hoc and proactive allocation was a rarity [59]. There is a need for a more assimilated oncology palliative care approach [20]. However, there appears to be a pre-conceived view that palliative care is only for the terminal phase of the disease. The HCP’s in palliative care services have the expertise, knowledge and skills to improve the quality of life for women when introduced at the correct time within the disease trajectory and in consultation with their oncologist. The relationships these women have with their health care providers significantly impact their overall well-being. Informational needs were often interconnected with psychological needs. Crucial to this is a requirement for shared decision-making imparting disease knowledge from a HCP perspective at timely junctures allowing time for women to make informed decisions of their care; this is a rarity in the care of MBC patients [50].

### 9.1. Implications for future research and practice

The findings from this QES demonstrate women with MBC are poorly understood and there are failings in the care of this cohort particularly in their psychological wellbeing and holistic care. What is urgently required from a practice perspective is an open transparent, person-centred care plan, devised via a collaborative approach between medical oncologists and women with MBC. Furthermore, when a woman is diagnosed with MBC an automatic referral for psychological support is necessary, to support women’s mental health during this period. Psychological support must be scaffolded on each individual women’s needs providing strategies to cope with living with a life limiting disease. In order to provide consistency and enhanced care for these women, an identified case worker should be instated to support women with MBC. These findings can be used to inform planning of ongoing care in an integrated manner, so the woman is supported and managed effectively.

Additionally primary quantitative follow up research around MBC is required to determine the pattern of care management across jurisdictions and results explored to determine facilitating factors in the provision of high-quality cost effective and timely care. Findings from this study can be used to inform the development of a questionnaire for this work. Or the development of a nurse led intervention study focusing on the provision of support, education and advocacy for women living with MBC.

## 10. Limitations of this study

This review has limitations. Firstly, only papers published in the English language were included. Although the search strategy was comprehensive and rigorous it is recognised that in the application of language limits, we are likely to have omitted some relevant international studies related to women’s experiences of MBC. It is also recognised that there are particular challenges in retrieving qualitative research because of poor indexing [10]. To address this additional search of supplementary sources such as grey literature, reference checking and hand searching were conducted.

## 11. Conclusion

It is clear from undertaking this QES the most significant unmet needs experienced by women with MBC, is a lack of holistic person-centred care, with no psychological support provided. It is evident that there is a dearth of knowledge pertaining to women’s mental health particularly after their initial diagnosis of MBC and coping with the oscillations of the disease. There is a requirement for Health Care Providers who care for these women to have training and education on supporting their holistic wellbeing. There is limited evidence of input from a multidisciplinary team perspective, and there appears to be no structured care pathway to ensure MBC patients received appropriate care and support [20, 44, 59]. Further research is required to explore the themes from this QES with women currently experiencing MBC and test the RAAW: Conceptual Framework for women living with MBC [adapted from ARC]. It is apparent that further research is urgently required to explore evidence based psychological interventions. The researchers of this QES feel there is merit in exploring a nurse led intervention study focusing on the provision of support, including psychological, education and advocacy for women living with MBC. The results of the Grade-CERQual indicate that there is high to moderate confidence in the majority of findings of this review. The result of this research adds to the available body of knowledge around women’s experiences of living with MBC and can inform developments in relation to integrated care models.

## Supporting information

S1 Table The seven-steps of ‘best fit’ framework. (ODT)

S2 Table SPICE framework. (ODT)

S3 Table Inclusion and exclusion criteria. (ODT)

S4 Table Search strings. (ODT)

S5 Table Characteristics of included studies. (ODT)

S6 Table CASP. (ODT)

S7 Table Summary of qualitative findings table. (DOCX)

S8 Table Enhancing the transparency in reporting the synthesis of qualitative research [ENTREQ]. (ODT)
