# Supplementary material for: Exploring the experiences of women living with metastatic breast cancer [MBC]: A systematic review of qualitative evidence
Source: PLoS One. 2024 Jan 5;19(1):e0296384. doi: 10.1371/journal.pone.0296384 (PMC10769043; doi:10.1371/journal.pone.0296384)
Supplement: S7 Table — (DOCX) [file pone.0296384.s007.docx]

S7 table: Summary of qualitative findings table

| **Summary of review finding** | **Studies contributing to the review finding** | **Methodological limitations** | **Coherence** | **Relevance** | **Adequacy** | **CERQual assessment of confidence in the evidence** | **Explanation of CERQual assessment** |
| --- | --- | --- | --- | --- | --- | --- | --- |
| **Finding 1**  **Receiving an initial diagnosis of MBC brought worry, fear, and an uncertain future into women’s lives.** | Alfieri et al [2022]; Bergqvist Strang [2017];  Chen et al. [2014];  Ginter [2020];  Krigel et al. [2014];  Lewis et al. [2016];  Lalayiannis et al. [2016];  Lundquist [2018];  Lundquist et al. [2020];  Maree & Mulonda [2015];  Mc Clelland [2015];  Lee Mortensen et al. [2018].  Mosher et al. [2013]  Reed & Corner [2015];  Schulman-Green et al. [2011]. | **Minor concerns** as five studies had minor issues concerning the bias in recruitment sample; one study had no ethical approval. 13 studies had no reference to reflexivity.  **12 No to very minor** **concerns** Alfieri et al [2022]; Bergvist Strang [2017]; Chen et al. [2014]; Ginter [2020]; Lalayiannis et al. [2016]; Lewis et al. [2016]; Lundquist [2018]; Mosher et al. [2013]; McClelland [2015] Krigel et al. [2014] Reed & Corner; [2015] Schulman-Green et al. [2011]  **2 minor concerns** Maree & Mulonda [2015]; Lundquist et al. [2020].  **1 minor to moderate** **concern** Lee Mortensen et al. [2018]. | **No concerns** | **No to very minor concerns regarding relevance concerns** Reduced global representation.  Six studies are from the USA, 2 UK 1 South Africa  1Canada 1 Sweden. Therefore, only one study from a developing country. | **No concerns** All were qualitative studies with a rich data set. | **High confidence** It is highly likely that the review finding is a reasonable representation of the phenomenon of interest. | **High confidence** in the review finding due to minor concerns regarding methodological limitations, no concerns regarding coherence, no to very minor concerns regarding relevance, no concerns in relation to adequacy. |

| **Summary of review finding** | **Studies contributing to the review finding** | **Methodological limitations** | **Coherence** | **Relevance** | **Adequacy** | **CERQual assessment of confidence in the evidence** | **Explanation of CERQual assessment** |
| --- | --- | --- | --- | --- | --- | --- | --- |
| **Finding 2**  **When women received a diagnosis of MBC, it impacted every aspect of their lives, highlighting a lack of psychological, emotional, and psychosocial support**. | Ahmad et al. [2012];  Alder et al. [2019];  Bergqvist Strang [2017]; Alfieri [2022]  Chen et al. [2014];  Ginter [2020];  Krigel et al. [2014];  Maree & Mulonda [2015];  Lee Mortensen et al. [2018];  Mosher et al. [2013];  Mosher et al. [2016]  Mc Clelland [2015];  Lalayiannis et al. [2016] ;  Lewis et al. [2015];  Lewis et al. [2016];  Lundquist [2018];  Lundquist et al. [2020];  Oostra et al. [2020];  Reed & Corner [2015]; Pacsi [2015]  Schulman-Green et al. [2011];  Smith et al. [2022]. | **Moderate concerns 1** study sample size was too small; only 3 participants, data analyses was not described. 1 study limited cohort interviewed, 1 study sample recruited via the same hospital as women with MBC received treatment and the researcher worked. 1 study had no ethical approval. Recruitment in 3 studies was flawed as there was minor recruitment bias. Only 2 studies referred to reflexivity.  **16 No to very minor** **concerns;**  Bergvist Strang [2017]; Chen et al. [2014]; Ginter [2020];  Krigel et al. [2014] Lalayiannis et al. [2016]; Lewis et al. [2015]; Lewis et al. [2016]; Lundquist [2018];Mosher et al. [2013]; Oostra et al. [2020]; Pacsi [2015]; Reed & Corner [2015] Schulman-Green et al. [2011]; Smith et al. [2022]: Alfieri et al. [2022];McClelland [2015];  **2 minor concerns :** Maree & Mulonda [2015] Lundquist et al. [2020].  **3 minor to moderate concerns:** Adler et al. [2019], Lee Mortensen et al. [2018]; Mosher et al. [2016].  **1 Moderate to serious Concerns** [Ahmad et al. 2011] | **Moderate concern**  Ahmad et al. [2012]. No description on how data was analysed. Focus on religion and cultural experiences not transferable to other cohorts of women globally.  Maree & Mulonda [2015] Education / understanding of MBC in third world setting is different and not transferrable to other parts of the world | **No to very Minor concerns**  Oostra et al. [2020] focused on nutritional problems in respect of women living with MBC. This was slightly off relevance because the focus was on particular nutritional issues and e-health rather than general experiences. | **No to very Minor concern** with Ahmad et al. [2012] study Only three women were included, therefore data thin. | **Moderate confidence** it is moderately likely that the finding is a reasonable representation of the phenomenon of interest. | **Moderate confidence in the review** **finding** due to moderate concerns regarding coherence, no to very minor concerns regarding relevance adequacy Moderate concerns regarding methodological limitations. |

| **Summary of review finding** | **Studies contributing to the review finding** | **Methodological limitations** | **Coherence** | **Relevance** | | **Adequacy** | **CERQual assessment of confidence in the evidence** | **Explanation of CERQual assessment** |
| --- | --- | --- | --- | --- | --- | --- | --- | --- |
| **Finding 3**  **Culture socio-economic standing influenced the availability of support for women with MBC.** | Adler et al. [2019] ;  Ahmad et al. [2011] ;  Maree & Mulonda [2015]:  Pacsi [2015] | **Moderate concern**  Only one study referred to reflexivity.  **No to very minor concerns: 1** Pacsi (2015) a small a purposive sample of self-identified second-generation, middle-class, married, Dominican American women this study cannot be generalized. **One minor** **concern** Maree & Mulonda [2015] Bias  **Minor to moderate** **concern; 1** Adler et al. [2019] Sample only required English speaking people to be eligible - There appears to be a conflict of interest. Only 3 participants were recruited, similar to the researcher’s background.    **Moderate to serious concerns;** 1 Ahmad et al. [2011]: Researcher was a volunteer with a support group. This study did not describe how the data was analysed. Literature utilised in the review was dated; research approach was not explained; No reference to ethical approval. | **Moderate concerns** in relation to the influence of cultural economic standing are evident within these four studies. Alder et al. [2019] did not describe how the data was analysed. Particular literature utilised in the review was dated; the research approach was not described in sufficient detail; In Ahmad et al. [2011] there was no reference to ethical approval. The researcher was a volunteer with a support group, there appears to be a conflict of interest. Only 3 participants were recruited who had similar backgrounds to the researcher. | | **Minor to Moderate**  **Concerns regarding relevance**  Within the study by Ahmed et al [2011] Literature is dated therefore less relevance to current experiences of women living with MBC. | **Moderate concerns** Maree & Mulonda [2015] study 7 of 10 had Stage IV breast cancer three women had grade 3B.  Ahmad et al. [2011] has only three participants  Only four studies supporting this finding. | **Low confidence** it is possible that the review finding is a reasonable representation of the phenomenon of interest | **Low confidence in the review finding due to** moderate concerns with Methodological limitations, moderate concerns in relation to coherence, moderate concerns with regard to relevance moderate concerns regarding relevance. |

| **Summary of review** | **Studies contributing to the review finding** | **Methodological limitations** | **Coherence** | **Relevance** | **Adequacy** | **CERQual assessment of confidence in the evidence** | **Explanation of CERQual assessment** |
| --- | --- | --- | --- | --- | --- | --- | --- |
| **Finding 4**  **Women with MBC experience a lack of information, knowledge MDT support. With evidence that models of care are no longer fit for purpose. This is different to a diagnosis of early breast cancer.** | Adler et al. [2019] ;  Ahmad et al. [2011] ; Alfieri et al [2022]  Bergvist & Strang [2017];  Chen et al. [2014];  Ginter [2022]; Greco [2022];  Kemp et al. [2018];  Lalayiannis et al. [2016];  Lundquist [2018] ;  Lewis et al. [2015];  Lewis et al. [2016];  Maree & Mulonda [2015] ;  Lee Mortensen et al. [2018] ;  Mosher et al. [2013]. Guite- Verret & Vachon [2021] ; Mc Clelland [2015]  Krigel et al. [2014];  Reed & Corner [2015]. | **Minor to Moderate concerns;** 1 study referred to reflexivity. 1 study had a limited cohort interviewed; interpreter not provided. 1 study did not describe how data was analysed. In one study, the sample was only of single women. In 1 study, women had to be proficient using social media; two studies bias via women having the ability to exercise, 1 study participants had to be able to engage with social media. 1 study had no ethical approval.  **15 No to very minor concerns;** Alfieri et al. [2022] ; Bergvist & Strang [2017[, Chen et al. [2014];Ginter [2022]; Greco [2022} Kemp et al. [2018] ; Lalayiannis et al. [2016]; Guite- Verret & Vachon [2021]. | **No concerns** | **No to very minor concerns**  Adler et al. [2019] study referred to a specific group of underserved women with MBC on the poverty line in the USA  Ahmad et al. [2011] study had too small a sample size of only three participants, these individuals had a similar background to the researcher. | **No to very minor** concerns, one study out of 16 studies had too few participants Ahmad et al. [2011]. | **Moderate confidence** It is moderately likely that the review finding is a reasonable representation. | **Moderate confidence** due to minor to moderate concerns with methodological limitations, no concerns in relation to coherence, no to very minor concerns regarding the relevance adequacy. |
| **Summary of review finding** | **Studies contributing to the review finding** | **Methodological limitations** | **Coherence** | **Relevance** | **Adequacy** | **CERQual assessment of confidence in the evidence** | **Explanation of CERQual assessment** |
|  |  | Lewis et al. [2015]; Lewis et al. [2016]; Lundquist [2018]; Mc Clelland [2015]  Mosher et al. [2013] ; Krigel et al. [2014]; Reed & Corner [2015].  **One minor concern** Maree & Mulonda [2015];    **Two minor to moderate** concerns Adler et al. [2019]; Lee Mortensen et al. [2018]  **One moderate to serious** Ahmad et al. [2011]. |  |  |  |  |  |

| **Summary of review finding** | **Studies contributing to the review finding** | **Methodological limitations** | **Coherence** | **Relevance** | **Adequacy** | **CERQual assessment of confidence in the evidence** | **Explanation of CERQual assessment** |
| --- | --- | --- | --- | --- | --- | --- | --- |
| **Finding 5**  **Women had difficulty talking about their diagnosis of MBC to family and friends. This caused a change in relationships emphasised the need/importance of social psychological support for this cohort.** | Adler et al. [2019]; Bergqvist & Strang [2017]; Chen et al. [2014];;Ginter [2020] ; Ginter [2022] ;Kemp et al. [2018]; Krigel et al. [2014]; Lalayiannis et al. [2016];  Lewis et al. [2015];  Lewis et al. [2016]; Lewis et al [2021] ; Lundquist [2018]; Lundquist et al. 2020]; Guite- Verret & Vachon [2021] | **Moderate concerns**  One study had a limited cohort interviewed, one study, the sample women with MBC, were all single reflexivity present in two studies.  **16 No to very minor concerns:** Bergvist & Strang [2017]; Chen et al. [2014]; Ginter [2020]; Ginter [2022] ; Kemp et al. [2014]; Krigel et al. [2014]; Lalayiannis et al. [2016]; Lewis et al. [2015]; Lewis et al. [2016]; Lewis et al. [2021] ; Lundquist [2018]; Mosher et al. [2013]; Mc Clelland [2015]; Pacsi [2015];Schulman-Green et al. [2011] Guite-Verret & Vachon [2021] | **No Concerns** | **No concerns** | **No concerns** | **Moderate confidence** It is moderately likely that the review finding is a reasonable representation of the phenomenon of interest. | Due to **moderate concerns** with methodological limitations, no concerns in relation to coherence, relevance or adequacy. |

| **Summary of review finding** | **Studies contributing to the review finding** | **Methodological limitations** | **Coherence** | **Relevance** | **Adequacy** | **CERQual assessment of confidence in the evidence** | **Explanation of CERQual assessment** |
| --- | --- | --- | --- | --- | --- | --- | --- |
|  | Maree & Mulonda [2015] ;  Mc Clelland [2015]; Mosher et al. [2013] Mosher et al [2016] ; Lee Mortensen et al. [2018] ; Pacsi [2015] ;  Schulman-Green et al. [2011] | **Three minors to moderate** concerns Adler et al. [2019]: Lee Mortensen et al. [2018]; Mosher et al [2016].  **Two minor concern** Maree & Mulonda [2015]: Lundquist et al. [2020] |  |  |  |  |  |

| **Summary of review finding** | **Studies contributing to the review finding** | **Methodological limitations** | **Coherence** | **Relevance** | **Adequacy** | **CERQual assessment of confidence in the evidence** | **Explanation of CERQual assessment** |
| --- | --- | --- | --- | --- | --- | --- | --- |
| **Finding 6**  **The impact of treatment and the array of debilitating symptoms from treatment had a detrimental effect on women’s quality of life ability to adjust.** | Adler et al. [2019]; Bergqvist & Strang [2017]; Chen et al. [2014]; ; Ginter [2020]; Greco [2022]; Krigel et al. [2014]; Kemp [2014] : Lalayiannis et al. [2016]; Lewis et al. [2015]; Lewis et al. [2016]; Lewis et al. [2021];Lundquist [2018]; Lundquist et al. [2020] McClelland [2015]; Lee Mortensen et al. [2018];Mosher et al. [2013]; Maree & Mulonda [2015];  Lundquist [2020]; Pacsi [2015]; Reed & Corner [2015]; Schulman-Green et al. [2011]; Guite- Verret & Vachon [2021]. | **Minor concerns**  1 study had limited cohort interviewed no interpreter provided to individuals that English is their 2^nd^ language. 2 studies had reflexivity. In 1 study sample, the women were all single.  One study sample, the participants, received 20 dollars for participating in the research. In 1 study sample was self-selected, Caucasian women mainly; 2 studies had bias re-recruitment from a physical exercise component. The sample participants were recruited from the same hospital where the researchers worked in one study.1 study women had to engage with social media to be part of the study. | **No concerns** | **No to very minor concerns** with regard to relevance Lundquist, [2018] topic of interest young mothers with advanced breast cancer as opposed to the general population of women with MBC. | **No concerns** | **High confidence** It is likely that the phenomenon of interest is a reasonable representation of the phenomenon of interest. | Due **to minor concerns** in relation to methodology limitations, no concerns in relation to coherence, no to very minor concerns in relation to relevance and no concerns with regard to adequacy |

| **Summary of review finding** | **Studies contributing to the review finding** | **Methodological limitations** | **Coherence** | **Relevance** | **Adequacy** | **CERQual assessment of confidence in the evidence** | **Explanation of CERQual assessment** |
| --- | --- | --- | --- | --- | --- | --- | --- |
|  |  | Limited to women with significant distress; No Follow-up questions regarding the experiences.  **17 No to very minor concerns:** Bergvist & Strang [2017]; Chen et al. [2014]; Ginter [2020]; Greco [2022]; Kemp et al. [2014]  Krigel et al. [2014]; Lalayiannis et al. [2016]; Lewis et al. [2015]; Lewis et al. [2016]; Lewis et al. [2021]; Lundquist [2018]; Mosher et al. [2013]; McClelland [2015]; Pacsi [2015] Reed & Corner [2015]; Schulman-Green et al. [2011]; Guite- Verret & Vachon [2021].  **Two minor concern** Maree & Mulonda [2015]; Lundquist et al. [2020].  **Three minor to moderate** **concerns** Adler et al. [2019];Lee Mortensen et al. [2018]; Mosher et al. [2013]. |  | . |  |  |  |

| **Summary of review finding** | **Studies contributing to the review finding** | **Methodological limitations** | **Coherence** | **Relevance** | **Adequacy** | **CERQual assessment of confidence in the evidence** | **Explanation of CERQual assessment** |
| --- | --- | --- | --- | --- | --- | --- | --- |
| **Finding 7**  **Living on reduced time was the catalyst for a substantial shift in changing lifestyles seeking deeper connections meaning within their lives. Having hope in the treatment they received, undertaking mindfulness, spirituality created short quantifiable goals; this aided the women to cope.** | Adler et al. [2019];  Bergqvist & Strang [2017];  Chen et al. [2014];  Ginter [2020] ;  Krigel et al. [2014];  Lalayiannis et al. [2016];  Lewis et al. [2015];  Lewis et al. [2016];  Lundquist [2018];  Lundquist et al. [2020];  Maree & Mulonda [2015];  Lee Mortensen et al. [2018];  Mosher et al. [2013];  Mc Clelland [2015];  Guo et al. [2022]; Pacsi [2015];  Schulman-Green et al. [2011]; Guite- Verret & Vachon [2021]. | **Moderate concerns**  1 study had no ethical approval. 1 study had limited cohort interviewed no interpreter provided to individuals that English was their 2nd language**.** In 1 study sample, the women were all single. Only 2 studies had reflexivity. Two studies had bias re-recruitment from a physical exercise component.  In 1 study, the participants had to have the ability to engage with social media to be part of the study; 1 study had a bias in relation to recruitment via the same hospital as participants were receiving treatment. In 1 study sample, the participanst received 20 dollars from participating in the research. The sample was self-selected in 2 studies. The majority of participants were Caucasian, married, well-educated. | **No concerns** | **No to minor concerns regarding relevance**  All data pertained to women living with breast cancer over the age of 18  Data was across the low middle high-income countries. | **No concerns** | **Moderate confidence** It is moderately likely that the review finding is a reasonable representation of the phenomenon of interest. | Due to **moderate concerns** in relation to methodological limitations, no concerns in relation to coherence, no to minor concerns in relation to relevance, no concerns with regard to adequacy. |

| **Summary of review finding** | **Studies contributing to the review finding** | **Methodological limitations** | **Coherence** | **Relevance** | **Adequacy** | **CERQual assessment of confidence in the evidence** | **Explanation of CERQual assessment** |
| --- | --- | --- | --- | --- | --- | --- | --- |
|  |  | **13 No to very minor concerns**  Bergvist & Strang [2017]; Chen et al. [2014]; Ginter [2020];  Krigel et al. [2014] Lalayiannis et al. [2016]; Lewis et al. [2015]; Lewis et al. [2016]; Lundquist [2018]; Mosher et al. [2013]; Mc Clelland [2015]; Pacsi [2015]; Schulman-Green et al. [2011]; Guite- Verret & Vachon [2021]  **Two minor concerns** Maree & Mulonda [2015]; Lundquist et al. [2020]  **Three minor to moderate** **concerns;** Adler et al. [2019]; Lee Mortensen et al. [2018]; Mosher et al. [2016] |  |  |  |  |  |

| **Summary of review finding** | **Studies contributing to the review finding** | **Methodological limitations** | **Coherence** | **Relevance** | **Adequacy** | **CERQual assessment of confidence in the evidence** | **Explanation of CERQual assessment** |
| --- | --- | --- | --- | --- | --- | --- | --- |
| **Finding 8**  **The relationship women with MBC had with their Doctor/ HCP/MDT was crucial to coping with the disease. There was evidence of lack of shared decision making, passive relationship with experts, & a need for palliative care oncology support.** | Alder et al. [2019];Alfieri et al.[2022]; Bergqvist Strang [2017]; Guité-Verret &Vachon [2021], Guo et al. [2022]; Oostra et al. [2020]; Pacsi [2015]; Krigel et al. [2014]; Lewis et al. [2015]; Maree & Mulonda [2015];McClelland [2015] ;Lee Mortensen et al. [2018]; Reed & Corner [2015]; Smith et al. [2021], Schulman-Green et al. [2011]; Smith et al [2022]. | **Minor concerns**  1 study had a limited cohort interviewed - three participants; no interpreter provided to individuals that English was their 2nd language**.** 1 study referred to patients’ reflexivity. 1 study had no ethical approval.  **13 No to very minor concerns** Alfieri et al. [2022]; Bergvist Strang [2017]; ; Guité-Verret & Vachon [2021]; Guo et al. [2022]; Krigel et al. [2014]; Lewis et al. [2015]; McClelland [2015] ;  Oostra et al. [2020] ; Pacsi [2015];  Reed & Corner [2015]. Schulman-Green et al. [2011]; Smith et al [2022];  **1 minor concern :** Maree & Mulonda [2015]  **2 minor to moderate** **concerns** Adler et al. [2019] Lee Mortensen et al. [2018]. | **No concerns** | **No to very minor concerns**  Bias in relations to recruitment of the participants via the same hospital where they were receiving treatment.  1 study had bias re-recruitment from a physical exercise standpoint. The women participants with MBC had to be able to engage with exercise. | **No concerns** | **High confidence** It is likely that the review finding is a reasonable representation of the phenomenon of interest. | Due to **minor concerns** in relation to methodological limitations, no concerns in relation to coherence no to very minor concerns in relation to relevance, no concerns in relation to adequacy. |

| **Summary of review finding** | **Studies contributing to the review finding** | **Methodological limitations** | **Coherence** | **Relevance** | **Adequacy** | **CERQual assessment of confidence in the evidence** | **Explanation of CERQual assessment** |
| --- | --- | --- | --- | --- | --- | --- | --- |
| **Finding 9**  **Women with MBC felt altruistic and were encouraged to support other women with cancer because of their personal experiences: They wanted to help other individuals improve their experience of living with metastatic breast cancer.** | Ahmad et al. [2011] ;  Adler et al. [2019];  Bergqvist Strang [2017];  Chen et al. [2014];  Ginter [2020];  Krigel et al. [2014];  Lalayiannis et al. [2016];  Lewis et al. [2015];  Lewis et al. [2016]; Lewis et al [2021] ;  Lundquist [2018] ;  Lundquist et al. [2020];  Maree & Mulonda [2015];  Mosher et al. [2013];  Mc Clelland [2015]  Pacsi [2015];  Schulman-Green et al. [2011]. | **Minor concerns**  1 study sample size is small, data analyses were not described. 1 study had limited cohort interviewed; no interpreter provided to individuals that English is their 2nd language**.** Only 2 studies had had evidence of researcher reflexivity; In 1 study participants were recruited via the hospital as receiving treatment. 1 study participants received $20 for their time in the form of a gift card mailed to.  **12 No to very minor concerns:**  Bergvist & Strang [2017]; Chen et al. [2014]; Ginter [2020]; Krigel et al. [2014]; Lalayiannis et al. [2016]; Lewis et al. [2015]; Lewis et al. [2016]; Lewis et al [2021]; Lundquist [2018]; Mosher et al. [2013]; Mc Clelland [2015] : Pacsi (2015);  Schulman-Green et al. [2011]  **2 minor concerns** Maree & Mulonda [2015]; Lundquist et al. [2020].  **1 minor to moderate** **concern** Adler et al. [2019]  **1 Moderate to serious** Ahmad et al. [2011] | **No Concerns** | **No to minor concerns**. 1 study Bias in relation to recruitment from a physical exercise component. One study Bias re-recruitment from a physical exercise component.1 study women had to engage with social media to be part of the study, Bias re-recruitment 1 study the sample primarily consisted of college-educated Caucasian patients. | **No Concerns** | **High confidence**: It is likely that the review finding reasonably represents the phenomenon of interest. | Due to **minor concerns** in relation to methodological limitations, no concerns in relation to coherence, no to minor concerns in relation to relevance mo concerns in relation to adequacy. |

| **Summary of review finding** | **Studies contributing to the review finding** | **Methodological limitations** | **Coherence** | **Relevance** | **Adequacy** | **CERQual assessment of confidence in the evidence** | **Explanation of CERQual assessment** |
| --- | --- | --- | --- | --- | --- | --- | --- |
| **Finding 10**  **Women living with MBC wanted to be identified as having a chronic illness; they went through a process of reinvention, creating a new future identity.** | Adler et al. [2019]; Ahmad et al. [2011]; Bergqvist Strang [2017]; Chen et al.[2014];]; Ginter [2020]; Greco [2022]; Krigel et al. [2014]; Lalayiannis et al. [2016]; Lewis et al. [2015]; Lewis et al.[2016]; Lundquist [2018]; Lundquist et al. [2020]; Maree & Mulonda [2015]; Lee Mortensen et al. [2018]; Mosher et al. [2013]; Pacsi [2015];Reed & Corner [2015]; Guite- Verret & Vachon [2021]. | **Minor concerns:** 1 study sample size is small, data analysis not described. 1 study had limited cohort interviewed; no interpreter provided to individuals that English is their 2nd language**.** Only 2 studies referred to reflexivity. In 1 study women had to engage with social media to be part of the study. 1 study had no ethical approval.  **14 No to very minor concerns**: Bergvist & Strang [2017]; Chen et al. [2014]; | **No concerns** | **No to very minor concerns** One study had a bias in relation to the recruitment of participants from a physical exercise component within the research. One study had bias concerning the recruitment of the sample through the same hospital the researchers worked as they were receiving treatment. One study sample was self-selected Caucasian women mainly. | **No concerns** | **High Confidence**: The review finding is likely a reasonable representation of the phenomenon of interest. | Due to **minor concerns** in relation to methodological limitations, no concerns in relation to coherence, no to very minor concerns in relation to relevance, no concerns in relation to adequacy. |

| **Summary of review finding** | **Studies contributing to the review finding** | **Methodological limitations** | **Coherence** | **Relevance** | **Adequacy** | **CERQual assessment of confidence in the evidence** | **Explanation of CERQual assessment** |
| --- | --- | --- | --- | --- | --- | --- | --- |
|  |  | Ginter [2020]; Greco [2022]; Krigel et al. [2014]; Lalayiannis et al. [2016]; Lewis et al. [2015]; Lewis et al. [2016]; Lundquist [2018]; Mosher et al. [2013]; Pacsi [2015]; Reed & Corner [2015]. Schulman-Green et al. [2011]; Guite- Verret & Vachon [2021].  **Two minor concerns:** Maree & Mulonda [2015] Lundquist et al. [2020]  **Two minor to moderate** **concerns:** Adler et al. [2019], Lee Mortensen et al. [2018]; |  |  |  |  |  |
