# Supplementary material for: Exploring the experiences of women living with metastatic breast cancer [MBC]: A systematic review of qualitative evidence
Source: PLoS One. 2024 Jan 5;19(1):e0296384. doi: 10.1371/journal.pone.0296384 (PMC10769043; doi:10.1371/journal.pone.0296384)
Supplement: Supplementary file 8 [file pone.0296384.s001.odt]

S1table: The seven steps of ‘best fit’ framework technique.

| Step 1 | Review question | What are the experiences of women living with metastatic breast cancer? |
| --- | --- | --- |
| Step 2 | Search of databases: Data bases were searched to identify relevant primary studies with the help of an information specialist; twenty-two studies were identified. | Simultaneously reviewed/ searched this literature for suitable models/theories/ framework – using BeHEMOTH template. ‘Best fit’ framework was identified |
| Step 3 | Extracted data from study characteristics CASP A quality appraisal was conducted using an adapted qualitative Critical Appraisal Skills Programme [CASP] checklist/tool | Best fit framework - A priori framework identified -The Adversity, Restoration, Compatibility [ARC] Conceptual framework for living with & beyond cancer, by Le Boutiller et al. [2019] was identified as the most suitable Framework |
| Step 4 | Evidence was coded from included studies against the ARC conceptual Framework within an adapted google form. |
| Step 5 | A black background with a black square  Description automatically generated with medium confidence Thematic synthesis was undertaken - new themes emerged |
| Step 6 | A black background with a black square  Description automatically generated with medium confidence New framework: RAAW; The Conceptual Framework for women living with MBC [adapted from ARC]. Reality & Adversity: A diagnosis of MBC; Adjustment: Living with MBC; Wellbeing: Awareness, meaning engagement [RAAW; MBC]. |
| Step 7 | A black background with a black square  Description automatically generated with medium confidence Assessing confidence in the review findings -The GRADE CERQual approach: GRAD CERqual methodological limitations tested in total ten findings from this review; five were deemed to have high confidence in the review findings, four were considered to have moderate confidence, one finding had a result of low confidence. |

Booth, A, Carroll, C. How to build up the actionable knowledge base: the role of "best fit" framework synthesis for studies of improvement in healthcare’, BMJ Quality & Safety, [2015]. 24[11], 700 –708. [http://doi.org/10.1136/bmjqs-2014-003642](http://doi.org/10.1136/bmjqs-2014-003642)
