# Supplementary material for: Exploring the experiences of women living with metastatic breast cancer [MBC]: A systematic review of qualitative evidence
Source: PLoS One. 2024 Jan 5;19(1):e0296384. doi: 10.1371/journal.pone.0296384 (PMC10769043; doi:10.1371/journal.pone.0296384)
Supplement: Supplementary file 9 [file pone.0296384.s002.odt]

S2 table: The SPICE Framework  

| Settings | Any setting: Hospital, oncology, palliative care units, home, community settings outpatients of Oncology/Palliative Care Units. |
| --- | --- |
| Population perspective | Women over the age of 18 diagnosed with MBC. |
| Intervention/phenomenon of interest | Women living with MBC. |
| Comparison | Potential comparison across age, geography/location, socio-economic factors, low high-income countries. |
| Evaluation | Attitudes, perceptions, and experiences, of living with metastatic breast cancer. |
