# Supplementary material for: Exploring the experiences of women living with metastatic breast cancer [MBC]: A systematic review of qualitative evidence
Source: PLoS One. 2024 Jan 5;19(1):e0296384. doi: 10.1371/journal.pone.0296384 (PMC10769043; doi:10.1371/journal.pone.0296384)
Supplement: Supplementary file 10 [file pone.0296384.s003.ODT]

| S3 table: Criteria for Inclusion and Exclusion. |
| --- |
| Criteria for selection | Included | Excluded |
| Types of research papers | Publication of primary research | Systematic, integrative & literature reviews, QES, & descriptive a research papers. |
| Types of studies | Qualitative studies on women’s experiences of living with MBC, with adult female population exploring the physical, psychological, emotional, practical aspects of an MBC diagnoses. Primary studies that use qualitative designs, e.g. case studies, phenomenology, ethnography, qualitative process evaluations & grounded theory studies. Primary qualitative studies that utilise qualitative research methods for data collection. Data analysis using qualitative methods, e.g. framework analysis, thematic analysis, grounded theory. Mixed or “hybrid” studies that include qualitative information on the experience’s perceptions, attitudes, understanding or comprehension of women living with metastatic breast cancer [collected by interview, focus group, case study or other qualitative methods]. Studies that use open text on a questionnaire with the data analysed qualitatively Qualitative studies looking at women diagnosed with breast cancer where the experience of those with metastatic disease is significant. Grey literature reports of dissertations/theses. Reported in English | Quantitative studies that data is collected utilising qualitative methods [e.g open-ended survey questions] Studies that concentrate on non-MBC /or cancer of the breast in men Studies with a paediatric population [children or adolescent] Individual’s male, females under the age of 18, Studies on other types of cancer experiences of individuals or/ with advanced chronic disease other than breast cancer Studies that are researching the biological medical management of the disease, although if the emphasis of the study is on the individual experience of treatment, these will be included. |
| Types of participants | Women >18yrs: diagnosed with MBC | Women <18yrs, Men, children |
| Types of Settings | Any setting hospital – oncology palliative care units, home  community settings |  |
| Types of Outcomes | Experiences, perception, attitude, views, opinion, belief, understanding, recognition, comprehension | Outcomes measured using quantitative methods |
| Time restrictions | 2010- 2022 | Any studies outside of this time limit |
