# Supplementary material for: Exploring the experiences of women living with metastatic breast cancer [MBC]: A systematic review of qualitative evidence
Source: PLoS One. 2024 Jan 5;19(1):e0296384. doi: 10.1371/journal.pone.0296384 (PMC10769043; doi:10.1371/journal.pone.0296384)
Supplement: Supplementary file 11 [file pone.0296384.s004.odt]

| # S4 table: Search Strings |
| --- |
| S1 “metastatic breast cancer” OR "advanced breast cancer" OR “stage 4 breast cancer" OR "secondary breast cancer" OR "incurable breast cancer" OR "chronic breast cancer" OR "terminal breast cancer” OR “life-limiting breast cancer diagnosis" OR "terminal breast cancer diagnosis" OR ‘Palliative care and metastatic breast cancer’ OR ‘metastatic breast tumour’ OR “metastatic breast tumor” OR "metastatic breast carcinoma." |
| OR |
| S2 “metastatic breast neoplasm” OR "Advanced breast neoplasm" OR "stage 4 breast neoplasm" OR "secondary breast neoplasm" OR "incurable breast neoplasm" OR "chronic breast neoplasm" OR "terminal breast neoplasm" OR "breast neoplasm" OR "life-limiting breast neoplasm diagnosis" OR "terminal breast neoplasm diagnosis" OR “recurrent breast neoplasm" OR “terminal breast neoplasm.” |
|  |
| S3 “Women’s* Experience* OR “perception” OR “perceive” OR “attitude*” OR “views” OR “opinion*” OR “belief” OR “Understanding” OR “recognition” OR “comprehension.” |
|  |
| S4 “Qualitative research” OR “qualitative study” OR “qualitative methods” OR “qualitative design” OR “mixed-method” OR “observation” OR “focus group*” OR Interview* OR Phenomen* OR hermeneutic* OR ethnography* OR “grounded theory” OR “thematic analysis” OR narrative OR “case study.” |
| Limits January 2010 – December 2022 Full text, peer-reviewed research papers English language |
