# Supplementary material for: Exploring the experiences of women living with metastatic breast cancer [MBC]: A systematic review of qualitative evidence
Source: PLoS One. 2024 Jan 5;19(1):e0296384. doi: 10.1371/journal.pone.0296384 (PMC10769043; doi:10.1371/journal.pone.0296384)
Supplement: Supplementary file 12 [file pone.0296384.s005.odt]

S5 table: Characteristics of included studies.

| Study No | Author | Year | Country | Design/Method | Sample | Analysis | Study focus |
| --- | --- | --- | --- | --- | --- | --- | --- |
|  | Adler et al. | [2019] | USA | A qualitative phenomenological study | 63 Women with MBC in receipt of low wage | Qualitative thematic analysis | This study focused on females with MBC who were inadequately provided with medical care in the community setting. |
|  | Ahmad et al. | [2011] | Malaysia | Qualitative descriptive | 3 Muslim women with MBC | Interviews were analysed using a constant comparative method | The focus of the study was to reveal the meaning of living with MBC via a narrative description, the role spirituality plays in coping with MBC for Muslim women. |
|  | Alfieri et al. | [2022] | Italy | Qualitative study | 9 women with MBC, between 36-74 years old | Semi-structured interviews were analysed using Braun Clark step by step process | The study focus was to achieve an in depth understanding of patients’ needs related to the MBC care pathway |
|  | Bergqvist & Strang | [2017] | Sweden | A qualitative study using semi-structured interviews | 20 women with MBC on at least 2nd -8th line of palliative chemotherapy | Seven steps of phenomenological analysis. Utilising a second-order perspective | The focus of this study was to explore the experiences of women with MBC on palliative chemotherapy. |
|  | Chen et al. | [2014] | Canada | A qualitative descriptive design | A purposive sample of 10 women aged 31-69. Living with MBC for three months - 4 years | Qualitative content analysis. | To investigate the changed functional ability of women socially with MBC |

| Study No | Author | Year | Country | Design/Method | Sample | Analysis | Study Focus |
| --- | --- | --- | --- | --- | --- | --- | --- |
|  | Ginter | [2020] | USA | A cross-sectional qualitative study. Phenomenology life course theory scaffolded the interviews. | Purposive sampling was utilised with nine women under the age of 41 years old diagnosed with MBC. | Phenomenological qualitative analysis using LCT to guide analysis. | To understand the lived experience of young women with a diagnosis of MBC. |
|  | Ginter | [2022] | USA | A cross-sectional qualitative study. Phenomenology life course theory scaffolded the interviews. | Purposive sampling of nine women. | Phenomenological qualitative analysis using LCT to guide analysis. | To understand how young women with MBC describe the lived experience of social and personal relationships after an MBC diagnosis |
|  | Greco | [2022] | UK & France | The comparison of experiences of patients in UK France with MBC in-depth interviews with patients with MBC | Interviewees contacted via medical institutions open ended semi- structured interviews with 10 patients with MBC | Exploration & impact of MBC diagnosis; the differences between the primary & secondary diagnosis & their relationship with medical professionals; MBC on everyday life. | Analyse how experiences & subjectivities of patients with MBC in the UK & France are characterised by complex uncertainties that cause life disruptions that are impossible to mend |

| Study No | Author | Year | Country | Design/Method | Sample | Analysis | Study Focus |
| --- | --- | --- | --- | --- | --- | --- | --- |
|  | Guité-Verret & Vachon | [2021] | Canada | Interpretative phenomenological analysis | 4 women living with MBC | IPA analysis | To better understand the experience of four women with incurable metastatic breast cancer the metaphors they used in personal cancer blogs |
|  | Guo et al. | [2022] | China | A hermeneutic phenomenological study | 20 Chinese women | Thematic analysis | The qualitative experiences & coping mechanisms of women with MBC undergoing chemotherapy in Beijing. |
|  | Kemp et al. | [2018] | Australia | Two qualitative studies. | Study [i] 21 women with MBC – telephone interviews  Study [ii] 15 women engaged with an online intervention | Thematic analysis Using framework method. | Study [i] focus was to address if women with MBC could be supported utilising an online forum to gain informational support [ii] was an online intervention specifically created for women with an early diagnosis of breast cancer beneficial for women with MBC. |
|  | Krigel et al. | [2014] | USA | Qualitative study using focus groups | A purposive sample of 15 women with A diagnosis of MBC. | Qualitative content analysis using Interpretive Phenomenology analysis. | To develop supportive interventions, & improve care for women with MBC |
|  | Lalayiannis et al. | [2016] | UK | A qualitative study | A purposive sample of 7 women living with secondary breast cancer. | Interpretive Phenomenological Analysis. | Exploring how women with MBC perceive/understand their illness, how they inform their teenage children [12-19 yrs.] of their prognosis. |

| Study No | Author | Year | Country | Design/Method | Sample | Analysis | Study Focus |
| --- | --- | --- | --- | --- | --- | --- | --- |
|  | Lee Mortensen et al. | [2018] | Denmark | Qualitative Study | 18 women were interviewed via five qualitative focus group interviews | Content analysis a constructivist approach. | The focus of the study was to explore the supportive requirements of health-related quality of life [HRQol] of females with MBC, long-term in Denmark. |
|  | Lewis et al. | [2015] | Australia | Qualitative approach | 18 females with were enrolled from a cross-sectional research project on the physical fitness of women with MBC. Semi-structured interviews were undertaken | Thematic analysis | The study focus: To ascertain informational support requirements of women with MBC. |
|  | Lewis et al. | [2016] | Australia | Qualitative social constructionist approach. | Semi-structured interviews with A purposive sample of 18 women living with MBC | Constructivist approach to thematic analysis | The study focus was on how women learn to live well with MBC. |
|  | Lewis et al | [2021] | Australia | Qualitative constructionist approach. | A purposive sample of 38 women living with MBC | Constructivist approach to thematic analysis | Aim: to explore how the experiences of women with MBC give meaning to choice in relation to their health. |
|  | Lundquist | [2018] | USA | A qualitative study [thesis] using a hermeneutic phenomenological approach | A purposive sample of 12 young women aged between 25-39yrs old with a diagnosis of MBC. | Van Manen method of manual coding reading transcripts repeatedly was undertaken. | The focus of the study is the meaning young women living with MBC [ages 25-39 years] give to everyday living. |

| Study No | Author | Year | Country | Design/Method | Sample | Analysis | Study Focus |
| --- | --- | --- | --- | --- | --- | --- | --- |
|  | Lundquist et al. | [2020] | USA | A qualitative study using a hermeneutic phenomenological approach | A secondary qualitative analysis of 21 semi-structured interviews of 12 young women living with MBC. Based on the theme ‘I am still a mom’ | Qualitative analysis of the theme ‘I’m still a mom’ using van Manen's hermeneutic phenomenology approach. | The focus of the study, was to glean a richer understanding of the experience of being a young mother living with MBC. |
|  | Maree & Mulonda | [2015] | South Africa | A descriptive qualitative design. | 10 women living with MBC | Thematic analysis | Zambian women’s experience of living with MBC |
|  | McClelland | [2015] | USA | A qualitative descriptive study. This study is part of a more extensive study relating to the quality of life of women diagnosed with MBC | 32 women diagnosed with MBC aged 35-77yrs old | Thematic analysis | The study focused on exploring women living with MBC descriptions of support they required to aid their sexual quality of life in palliative care. |
|  | Mosher et al. | [2013] | USA | A qualitative study Trial design was published previously | 44 women completed expressive writing essays | Theoretical thematic analysis coding of 176 essays | To identify concerns amongst distressed women with MBC & to highlight complex, physical, psychological anguish & practical considerations. |

| Study No | Author | Year | Country | Design/Method | Sample | Analysis | Study Focus |
| --- | --- | --- | --- | --- | --- | --- | --- |
|  | Mosher et al. | [2016] | USA | Qualitative study | 25 women with stage IV breast cancer were interviewed using semi-structured interviews | Thematic analysis | Explore women with MBC perceptions of symptom importance |
|  | Oostra et al. | [2020] | USA | An exploratory qualitative study | Semi-structured interviews were undertaken with 21 women with a diagnosis of MBC on an eHealth platform | Team-based Content analysis | To qualitatively assess the dietary concerns of women with MBC & explore ways to address these problems with an e-Health platform |
|  | Pacsi | [2015] | USA | Descriptive phenomenological study | A purposive sample of 6 American women of Dominican descent living with MBC. | The data was analysed using Giorgi’s [1999, 2005] 5 step human scientific method | Gain deeper insight into a subgroup of Hispanics living with MBC |

| Study No | Author | Year | Country | Design/Method | Sample | Analysis | Study Focus |
| --- | --- | --- | --- | --- | --- | --- | --- |
|  | Reed & Corner | [2015] | UK | Longitudinal mixed methods study. 235 women were recruited for a national questionnaire survey results are reported elsewhere. Ten women from the two hundred thirty-five consented to participate in this longitudinal study. | 10 women with MBC aged 40 -78yrs of age. 8 women were Interviewed three times & 2 women were interviewed twice over 12 months using a qualitative approach | Holistic content analysis approach. | Investigate the illness trajectory of MBC using a framework of chronic illness model |
|  | Schulman-Green | [2011] | USA | An exploratory qualitative study. | Semi-structured interviews were undertaken of a purposive sample of 15 women with MBC | Coding analysis of Concepts themes | To describe self-management preferences & practices of women with MBC during transitions within the disease trajectory. |
|  | Smith et al. | [2022] | Australia | Interpretative social constructionist approach | Semi-structured interviews with 38 women living with MBC. | A constructivist approach to thematic analysis | Explore women’s experience of supportive care from breast care nurses |
