# Supplementary material for: Exploring the experiences of women living with metastatic breast cancer [MBC]: A systematic review of qualitative evidence
Source: PLoS One. 2024 Jan 5;19(1):e0296384. doi: 10.1371/journal.pone.0296384 (PMC10769043; doi:10.1371/journal.pone.0296384)
Supplement: Supplementary file 13 [file pone.0296384.s006.odt]

S6 table: Critical Appraisal Skills Programme [CASP] checklist/tool

| Study ID | Was the context described? | Was the sampling strategy appropriate & described? | Was the data collection strategy appropriate & described? | Was the data analysis appropriate &  described? | Were the findings supported by evidence? | Is there evidence of researcher reflexivity? | Have ethical issues been taken into consideration? | Overall assessment of methodological limitations |
| --- | --- | --- | --- | --- | --- | --- | --- | --- |
| Adler et al. 2019 | ü | û | ü | ü | ü | û | ü | Minor to moderate concerns |
| Ahmad et al. 2011 | ü | û | ü | û | ü | û | û | Moderate to serious concerns |
| Alfieri et al. 2022 | ü | ü | ü | ü | ü | û | ü | No to very minor concerns |
| Bergqvist & Strang 2017 | ü | ü | ü | ü | ü | û | ü | No to very minor concerns |
| Chen et al. 2014 | ü | ü | ü | ü | ü | û | ü | No to very minor concerns |
| Ginter 2020 | ü | ü | ü | ü | ü | û | ü | No to very minor concerns |
| Ginter 2022 | ü | ü | ü | ü | ü | û | ü | No to very minor concerns |
| Greco 2022 | ü | ü | ü | û | ü | û | ü | No to very minor concerns |
| Guité-Verret  Vachon 2021 | ü | ü | ü | ü | ü | ü | ü | No to very minor concerns |
| Guo et al. 2022 | ü | ü | ü | ü | ü | ü | ü | No to very minor concerns |

| Study ID | Was the context described? | Was the sampling strategy appropriate & described? | Was the data collection strategy appropriate & described? | Was the data analysis appropriate & described? | Were the findings supported by evidence? | Is there evidence of researcher reflexivity? | Have ethical issues been taken into consideration? | Overall assessment of methodological limitations |
| --- | --- | --- | --- | --- | --- | --- | --- | --- |
| Kemp et al. 2018 | ü | ü | ü | ü | ü | û | ü | No to very minor concerns |
| Kringel et al. 2014 | ü | ü | ü | ü | ü | û | ü | No to very minor concerns |
| Lalayiannis et al.  2016 | ü | û | ü | ü | ü | ü | ü | No to very minor concerns |
| Lee Mortensen et al. 2018 | ü | ü | ü | ü | ü | û | û | Minor to moderate concerns |
| Lewis et al 2015 | ü | û | ü | ü | ü | û | ü | No to very minor concerns |
| Lewis et al. 2016 | ü | û | ü | ü | ü | û | ü | No to very minor concerns |
| Lewis et al  2021 | ü | ü | ü | ü | ü | û | ü | No to very minor concerns |
| Lundquist 2018 | ü | ü | ü | ü | ü | û | ü | No to very minor concerns |
| Lundquist et al. 2020 | ü | û | ü | ü | ü | û | ü | Minor concerns |
| Maree  Mulonda 2015 | ü | û | ü | ü | ü | û | ü | Minor concerns |

| Study ID | Was the context described? | Was the sampling strategy appropriate & described? | Was the data collection strategy appropriate & described? | Was the data analysis appropriate & described? | Were the findings supported by evidence? | Is there evidence of researcher reflexivity? | Have ethical issues been taken into consideration? | Overall assessment of methodological limitations |
| --- | --- | --- | --- | --- | --- | --- | --- | --- |
| Mosher et al. 2016 | ü | û | ü | ü | ü | û | ü | Minor to moderate concerns |
| Mc Clelland 2015 | ü | û | ü | ü | ü | û | ü | Minor concerns |
| Mosher et al. 2013 | ü | ü | ü | ü | ü | û | ü | No to very minor concerns |
| Oostra et al. 2020 | ü | ü | ü | ü | ü | û | ü | No to very minor concerns |
| Pacsi 2015 | ü | ü | ü | ü | ü | ü | ü | No to very minor concerns |
| Reed & Corner 2015 | ü | ü | ü | ü | ü | û | ü | No to very minor concerns |
| Schulman-Green et al. 2011 | ü | ü | ü | ü | ü | û | ü | No to very minor concerns |
| Smith et al. 2022 | ü | ü | ü | ü | ü | û | ü | No to very minor concerns |
