# Supplementary material for: Exploring the experiences of women living with metastatic breast cancer [MBC]: A systematic review of qualitative evidence
Source: PLoS One. 2024 Jan 5;19(1):e0296384. doi: 10.1371/journal.pone.0296384 (PMC10769043; doi:10.1371/journal.pone.0296384)
Supplement: Supplementary file 14 [file pone.0296384.s008.odt]

| S8 table: Enhancing transparency in reporting the synthesis of qualitative research: [ENTREQ] Tong A., Flemming, K., McInnes, E., Oliver, S., Craig, J. Enhancing transparency in reporting the synthesis of qualitative research: ENTREQ statement.  BMC Medical Research Methodology, [2012] 12:181 [http://www.biomedcentral.com/1471-2288/12/181](http://www.biomedcentral.com/1471-2288/12/181) |
| --- |
| Item | Guide and description | Description on page |
| Aim | State the research question the synthesis addresses. | What are the attitudes, perspectives and experiences of women living with metastatic breast cancer? |
| Synthesis methodology | Identify the synthesis methodology or theoretical framework which underpins the synthesis, and describe the rationale for the choice of methodology (e.g. met- ethnography, thematic synthesis, critical interpretive synthesis, grounded theory synthesis, realist synthesis, meta-aggregation, meta-study, framework synthesis) | 3.0 Method Best fit framework synthesis method allows testing, reinforcement and building on an existing published model, for a similar population. |
| Approach to searching | Indicate whether the search was pre-planned (comprehensive search strategies to seek all available studies) or iterative (to seek all available concepts until theoretical saturation is achieved). | 3.2 Search strategy - pre-planned |
| Inclusion criteria | Specify the inclusion/exclusion criteria (e.g. in terms of population, language, year limits, type of publication, study type). | Table 3 Inclusion/exclusion criteria. |
| Data sources | Describe the information sources used. | 3.2 Search Strategy. |
| Item | Guide and description | Description on page |
| Electronic Search strategy | Describe the literature search (e.g., provide electronic search strategies with population terms, clinical or health topic terms, experiential or social phenomena related terms, filters for qualitative research and search limits). | Table 4 Search Strings |
| Study screening methods | Describe the process of study screening and sifting (e.g. title, abstract and full-text review, number of independent reviewers who screened studies) | 3.1 Systematic identification of primary studies 3.2. Search Strategy 3.3. Quality appraisal of included studies. 3.4. Selection of a framework for ‘best fit’ analysis; 4. ‘Best Fit’ Framework Synthesis 4.1. The ARC Conceptual Framework 4.2. Data extraction 4.3 Data synthesis |
| Study characteristics | Present the characteristics of the included studies (e.g. year of publication, country, population, number of participants, data collection, methodology, analysis, research questions). | Table 5 Characteristics of included studies. |
| Study selection Results | Identify the number of studies screened and provide reasons for study exclusion (e.g. for comprehensive searching, provide numbers of studies screened and reasons for exclusion indicated in a figure/flowchart; for iterative searching, describe reasons for study exclusion and inclusion based on modifications t the research question and/or contribution to theory development) | Fig 2 PRISMA Flow Diagram |
| Rationale for appraisal | Describe the rationale and approach used to appraise the included studies or selected findings (e.g.assessment of conduct (validity and robustness), assessment of reporting (transparency), assessment of content and utility of the findings). | 3.3 Quality appraisal of included studies. Table 6 CASP Table 7 Summary of qualitative findings table. |

| Item | Guide and description | Description on page |
| --- | --- | --- |
| Appraisal items | State the tools, frameworks and criteria used to appraise the studies or selected findings (e.g. Existing tools: CASP, QARI, COREQ, Mays and Pope [25]; reviewer developed tools; describe the domains assessed: research team, study design, data analysis and interpretations, reporting). | 3.3 Quality appraisal of included studies. Table 6 CASP Table 7 Summary of qualitative findings table |
| Appraisal process & results | Indicate whether the appraisal was conducted independently by more than one reviewer and if consensus was required. | 3.3 Quality appraisal of included studies. Table 6 CASP |
| Data extraction | Indicate which sections of the primary studies were analysed and how were the data extracted from the primary studies? | 4.2 Data extraction |
| Software | State the computer software used if any. | Covidence systematic review software. EndNote X9 (2020) Clarivate Analytics, PA, USA Google forms |
| Number of reviewers | Identify who was involved in coding and analysis. | Five reviewers: TLR, AC, PM, EC, EM. |

| Item | Guide and description | Description on page |
| --- | --- | --- |
| Study comparison | Describe how were comparisons made within and across studies (e.g. subsequent studies were coded into pre-existing concepts, and new concepts were created when deemed necessary). | 3.4 Selection of a framework for ‘best fit’ analysis, 4 ‘Best Fit’ Framework Synthesis 4.1 The ARC Framework. 4.2 Data extraction 4.3 Data synthesis |
| Derivation of themes | Explain whether the process of deriving the themes or constructs was inductive or deductive. | 4.2 Data extraction Table 1 The 7 steps of ‘best-fit framework’ |
| Synthesis output | Present rich, compelling and useful results that go beyond a summary of the primary studies (e.g. new interpretation, models of evidence, conceptual models, analytical framework, development of a new theory or construct). | Fig 4 RAAW: The Conceptual Framework for women living with MBC (adapted from ARC). Reality & Adversity: A diagnosis of MBC; Adjustment: Living with MBC; Wellbeing: Awareness, meaning and engagement (RAAW; MBC) Table 7 Summary of qualitative findings table. |
